# Cross-sectional and prospective associations between jump performance and functional outcomes in older adults: a systematic review and meta-analysis

**DOI:** 10.1186/s12877-026-07450-6

**Published:** 2026-04-11

**Authors:** Christian Werner, Anna Schumacher, Sandra Lau, Andrea Heinks, Rebecca Diekmann, Phoebe Ullrich, Jürgen M. Bauer

**Affiliations:** 1 Geriatric Center, Medical Faculty Heidelberg, Heidelberg University, Heidelberg, Germany; 2 Department of Health Services Research, Assistance Systems and Medical Device Technology, School VI – Medicine and Health Sciences, Carl von Ossietzky University Oldenburg, Oldenburg, Germany; 3 Department of Health Services Research, School of Medicine and Health Science, Carl von Ossietzky University Oldenburg, Oldenburg, Germany; 4 Department of Health Services Research, Nutrition and Physical Function in Older Adults (Junior Research Group), School VI – Medicine and Health Sciences, Carl von Ossietzky University Oldenburg, Oldenburg, Germany; 5 Department of Medical Oncology, Thorax Clinic, Heidelberg University Hospital, Heidelberg, Germany

**Keywords:** Aging, Physical examination, Fitness Testing, Muscle strength, Locomotion, Activities of daily living, Review

## Abstract

**Background:**

Early identification of age-related functional decline is essential, yet commonly used mobility assessments often lack sensitivity to detect early decline in high-functioning older adults. Muscle power deteriorates early with aging and has been associated with functional outcomes, suggesting it may be a sensitive marker of functional decline. Jump tests directly assess lower-limb muscle power, but their relationship with functional status or future outcomes has not been synthesized. This review evaluates cross-sectional and prospective associations between jump performance (JP) and functional outcomes in older adults.

**Methods:**

A systematic search of PubMed, Cochrane Library, IEEE Xplore, and PEDro was conducted from inception to 18 September 2025. Reference lists were also screened. Eligible studies included older adults (mean age ≥ 65 years), assessed JP using technological devices, and examined cross-sectional or prospective associations with functional outcomes. Risk of bias was assessed using Joanna Briggs Institute checklists. Random-effects meta-analyses were conducted when feasible; otherwise, narrative synthesis was performed.

**Results:**

Thirty-two studies (7794 participants; 73 ± 5 years) were included, with 16 contributing to meta-analyses. Most studies comprised high-functioning individuals. JP was primarily assessed using countermovement jumps on force platforms (28/32). Functional outcomes included locomotor capacity (LC), physical activity (PA), activities of daily living (ADL), falls, sarcopenia, frailty, and dysmobility syndrome. Methodological quality was predominantly moderate to high (29/32). Meta-analyses were feasible only for cross-sectional associations with LC (544–2519 participants per outcome). Higher jump power showed moderate associations with better performance in Timed Up and Go, gait speed, 5-Chair Stand Test, and Short Physical Performance Battery (|*r*_*pooled*_|= 0.36–0.48). Similar associations were found for jump height (|*r*_*pooled*_|= 0.26–0.40) and jump velocity (|*r*_*pooled*_|= 0.39–0.48). Between-study heterogeneity was predominantly high (I^2^ > 75% in most analyses; range 38.2–97.5%). Narrative synthesis showed associations of higher JP with greater PA, better ADL, and lower odds of sarcopenia and dysmobility syndrome, but not fall history. Longitudinal evidence was limited to two studies, suggesting prospective associations with falls but not incident (pre-)frailty.

**Conclusions:**

JP shows consistent moderate associations with LC in high-functioning older adults, suggesting potential value as a sensitive marker of early mobility decline. Evidence for associations with other functional outcomes and for prospective associations remains limited, highlighting the need for further studies, particularly with longitudinal designs.

**Trial Registration:**

Prospectively registered in PROSPERO (CRD42020207540).

**Supplementary Information:**

The online version contains supplementary material available at 10.1186/s12877-026-07450-6.

## Background

Aging is accompanied by a progressive decline in functional ability, affecting essential mobility-related activities of daily living (ADL) such as walking, rising from a chair, and climbing stairs [1–3]. Mobility, defined as the ability to be mobile, is crucial for independence, participation, and quality of life in older adults [4–6]. However, the ongoing demographic shift toward older populations in many Western countries is expected to substantially increase the prevalence of mobility, and consequently, functional limitations, posing significant challenges for healthcare systems and society. To enable preventive and timely interventions before substantial functional decline occurs, effective and feasible assessment methods for detecting individuals at risk of mobility decline at an early stage are essential. However, early detection remains challenging, partly because mobility decline is multifactorial in nature [7, 8], and no single assessment method has emerged as a definitive gold standard for assessing mobility or detecting early-stage mobility decline [9–11].

Commonly used tools for assessing older adults’ locomotor capacity, referring to performance-based mobility measures conducted under standardized conditions, such as the Short Physical Performance Battery (SPPB) [12, 13], the Performance Oriented Mobility Assessment [14, 15], and the Berg Balance Scale [13, 14] suffer from ceiling effects when applied in high-functioning, community-dwelling older adults. These limitations reduce their ability to detect early mobility deficits and subtle changes over time. Other widely used tools, including the Timed Up and Go (TUG) or the 5-Chair Stand Test (5CST), have likewise been considered insufficiently sensitive for detecting subtle mobility impairments [16] and may not be challenging enough to identify risk factors for adverse health outcomes such as falls in high-functioning older adults [17–19]. Although additional tools specifically developed for this population, such as the Community Balance and Mobility Scale [20] or Fullerton Advanced Balance Scale [21] offer greater challenges, their long administration time (10–30 min) limits their practical feasibility.

Muscle power, defined as the product of muscular force and velocity of movement, has been associated with future adverse health outcomes in older adults, including ADL disability, frailty, hospitalization, and mortality [22, 23]. Lower-limb muscle power also appears to be a stronger determinant of functional dependency [24] and mobility impairment [25, 26] than muscle strength. Importantly, lower-limb muscle power deteriorates earlier in the aging process than muscle strength [27], and it also tends to decline at a faster rate than both muscle strength and performance-based mobility measures such as gait speed and TUG [1, 27]. Given its close link to functional ability and its accelerated age-related decline, muscle power may serve as a particularly sensitive marker of early mobility decline in older adults [28].

Lower-limb muscle power in older adults can be assessed directly using laboratory-based methods such as vertical jump tests on force platforms, pneumatic resistance devices, unloaded leg extensor power rigs, linear position transducers, and isokinetic dynamometry [29, 30]. Functional power assessments, including sit-to-stand or stair-climb power tests [31, 32], also provide indirect estimates of lower-limb power during everyday movements. Jump tests combine elements of functional and muscle testing by assessing a high-intensity, multi-joint movement that relies not only on muscle force and power but also on other functional systems (e.g., neuromuscular, sensory) relevant for ADL [33]. Compared to sit-to-stand power tests, jump tests have been suggested to be more sensitive to age-related changes in muscle power [34]. Despite their explosive nature, jump tests have been shown to be safe for healthy older adults and demonstrate good reproducibility with minimal learning effects [33, 35]. Nevertheless, the feasibility of jump testing may be limited in lower-functioning older individuals, as successful completion requires a sufficient level of physical functioning. Consequently, jump tests may be most applicable in higher-functioning older adults and may be particularly useful for detecting early functional decline before more pronounced impairments occur. However, the clinical relevance of jump tests and specific jump parameters (e.g., power, height, velocity) for functional status or decline in older adults remains unclear, and no systematic review has yet synthesized the available evidence on this topic.

The aim of this systematic review and meta-analysis is to evaluate the cross-sectional and prospective associations between jump performance and functional outcomes in older adults, including multiple domains of functional and related health outcomes.

## Methods

This systematic review and meta-analysis followed the Preferred Reporting Items for Systematic reviews and Meta-Analyses (PRISMA) guidelines [36] and was registered in the International Prospective Register of Systematic Reviews (PROSPERO) database in October 2020 (CRD42020207540; clinical trial number: not applicable). Methodological adjustments made after registration included: (1) refining the age criterion to include studies with a mean participant age of ≥ 65 years (previously ≥ 60 years) to ensure a clearer focus on older adults; (2) expanding the scope to examine both the cross-sectional and prospective associations between jump performance and functional outcomes, providing a more comprehensive insight into its clinical relevance in older adults; (3) conducting meta-analyses where sufficient homogeneous data were available, although only qualitative synthesis had originally been planned; and (4) assessing risk of bias (RoB) using Joanna Briggs Institute (JBI) critical appraisal checklists instead of the originally planned Cochrane tool, as these were more appropriate for the predominantly observational study designs included in this review.

### Information sources and search strategy

A systematic literature search of four databases (PubMed, Cochrane Library, IEEE Xplore, PEDro) was conducted from inception to 18 September 2025. Full search strategies are provided in Table 1. The search was not restricted by publication date. The reference lists of all included studies and one relevant systematic review [37] were also hand-searched to identify additional eligible studies.

**Table 1:** Detailed search strategy for each bibliographic database

**PubMed**	**Cochrane Library**	**IEEE Xplore**	**PEDro**
#1 aged[MeSH] #2 "older persons" #3 "older adults" #4 elder* #5 seniors #6 geriatr* #7 #1 OR #2 OR #3 OR #4 OR #5 OR #6 #8 jump* #9 #7 AND #8 #10 suicid* #11 #9 NOT #10	#1 aged[MeSH] #2 "older persons" #3 "older adults" #4 elder* #5 seniors #6 geriatr* #7 #1 OR #2 OR #3 OR #4 OR #5 OR #6 #8 jump* #9 #7 AND #8	#1 "older persons" #2 "older adults" #3 elder* #4 seniors #5 geriatr* #6 #1 OR #2 OR #3 OR #4 OR #5 #7 jump* #8 #6 AND #7	#1 abstract & title: jump* #2 subdiscipline: gerontology #3 method: clinical trial #4 #1 AND #2 AND #3

### Eligibility criteria

Cross-sectional and longitudinal studies were eligible for inclusion if they (1) included older adults with a mean age ≥ 65 years, (2) measured jump performance using technological devices, (3) analyzed associations between jump performance and functional outcomes assessed concurrently or prospectively, and (4) were published in English or German. Studies in which jumping was used solely as an intervention or was examined in the context of suicidal behavior were excluded. Functional outcomes were defined as measures of locomotor capacity, functioning in ADL, physical activity (PA), and other function-related health outcomes such as falls, sarcopenia, and frailty.

### Study selection and data extraction

All identified records were imported into EndNote X9 (Clarivate Analytics, Philadelphia, PA, USA) for duplicate removal. After duplicates were removed, titles and abstracts were independently screened by two reviewers using Rayyan (Qatar Computing Research Institute, Doha, Qatar), a non-commercial web-based application for systematic review management [38]. Any disagreements were resolved through discussion and, if no consensus could be reached, through consultation with a third reviewer. The same procedure was applied to the full-text screening of the remaining potentially eligible studies. When multiple records reported results from the same study sample, the most comprehensive or recent report providing the relevant outcome data was retained, and earlier publications were excluded. When studies reported cross-sectional associations at multiple time points, the earliest (baseline) estimate was extracted to ensure comparability across studies.

Data extraction was performed by two reviewers using standardized data entry form that captured the following information: (1) study characteristics (full reference and country of origin); (2) methodological details (study design, sample size, proportion of women, mean age, and key sample characteristics); (3) assessment of jump performance (type of jump test, technological device or system used, and the specific jump parameters measured); (4) the functional outcome category and specific measure examined in relation to jump performance; and (5) findings related to cross-sectional and/or prospective associations between jump parameters and functional outcomes.

Unadjusted and adjusted associations were extracted, and adjusted associations were included irrespective of the specific covariates used in the model. If studies reported results for multiple age groups, only data from the age group with a mean age ≥ 65 years were extracted and included in the analyses. No study authors were contacted to obtain missing data or clarify study details.

### Risk of bias assessment

The methodological quality and RoB of the included studies were evaluated using the JBI Critical Appraisal Checklists. The JBI Checklist for Analytical Cross-sectional Studies was applied to all cross-sectional studies and to the cross-sectional components of mixed-design studies [39, 40]. For longitudinal components and longitudinal studies, the JBI Checklist for Cohort Studies was used [39, 40]. For studies contributing data to both cross-sectional and prospective analyses, RoB was assessed separately for each analysis using the appropriate JBI checklist. Each JBI item was scored as 1 for “yes” and 0 for “no” or “unclear”. An overall quality score for each study was calculated as the percentage of checklist items rated “yes” and classified as low (< 50%), moderate (50–69%), or high (≥ 70%) methodological quality. RoB assessment was conducted by one reviewer and independently verified by a second reviewer, with discrepancies resolved through discussion. RoB assessment did not influence study eligibility.

### Data synthesis and analysis

Associations between jump performance and functional outcomes were synthesized using both meta-analytic and narrative approaches. Meta-analyses were conducted when at least three studies reported statistically comparable associations for the same jump parameter and functional outcome, to support the robustness and interpretability of pooled estimates. Bivariate Pearson correlation coefficients (*r*) were used as the primary effect size for pooling. When required and feasible, other reported statistics were transformed to Pearson’s *r* prior to pooling. Spearman *ρ* was converted using Fisher’s approximation (*r* = 2·sin(π·ρ/6)) [41]. Unadjusted regression-based effect sizes, including coefficients of determination (R^2^), were converted using *r* = *√R*^*2*^*,* with the sign determined by the direction of association described in the text or figures. When studies reported separate correlations for men and women, subgroup-specific *r*-values were transformed using Fisher’s z, pooled using sample-size weighting, and back-transformed to a single overall *r*. All Pearson correlation coefficients, whether reported directly or derived, were then transformed using Fisher’s z for meta-analytic pooling and back-transformed to *r* for interpretation. Correlations were interpreted as low (|*r*|) < 0.30), moderate (0.30 ≤|*r*|) < 0.50), or high (|*r*|) ≥ 0.50) [42]. Random-effects meta-analyses were performed using Hartung-Knapp-Sidik-Jonkman methods [43], with between-study variance (*τ*^*2*^) estimated by the Paule-Mandel estimator [44]. Statistical significance was set at *p* < 0.05. Between-study heterogeneity was quantified using the *I*^*2*^ statistic and interpreted as low (~ 25%), moderate (~ 50%), or high (~ 75%) [45]. A subgroup analysis was performed to assess differences in pooled associations between jump height and locomotor capacity according to the device used to assess jump height (non-mechanography devices vs. force platforms), with differences evaluated using a random-effects Q-test. Publication bias and small-study effects were planned to be assessed using funnel plots and Egger’s test [46]; however, these analyses were not conducted due to the limited number of studies per meta-analysis (range: 3–6 studies). All meta-analyses were conducted in RStudio (version 2025.09.2, Build 418) using the *meta* package [47], specifying Fisher’s z–transformed correlations for computation. Results shown in forest plots represent the corresponding back-transformed correlation coefficients (*r*). When meta-analyses were not feasible, a narrative synthesis was conducted to provide a qualitative summary of findings. This applied to cross-sectional analyses reporting adjusted correlation or regression coefficients or odds ratios (OR) with heterogeneous adjustment sets or functional outcome definitions, and to longitudinal analyses that differed in outcome measures, included confounders, or follow-up duration.

## Results

### Literature search

**Fig. 1:**
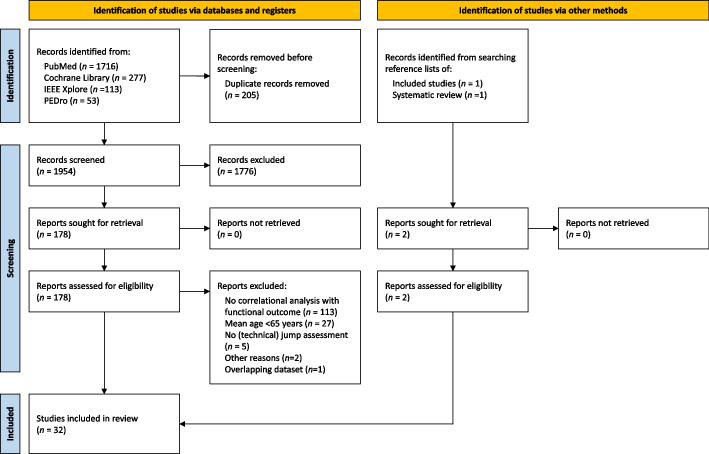
PRISMA flow chart of study inclusion and exclusion

A total of 2,159 studies were identified from PubMed (*n* = 1,716, 79.5%), Cochrane Library (*n* = 277, 12.8%), IEEE Xplore (*n* = 113, 5.2%), and PEDro (*n* = 53, 2.5%). After removing 205 duplicates (9.5%), 1,954 studies (90.5%) remained for title and abstract screening. Of these, 178 studies (9.1%) were retrieved and assessed for eligibility in full text. In total, 30 studies identified through database searches met the inclusion criteria [48–77]. Screening the reference lists of these studies and a relevant systematic review yielded 2 additional eligible studies [78, 79]. Ultimately, 32 studies reporting cross-sectional and/or prospective associations between jump parameters and functional outcomes in older adults were included in this review (Fig. 1).

### Study characteristics

An overview of the characteristics of all 32 included studies is presented in Supplementary Table S1 [48–79]. The articles were published between 1999 [59] and 2025 [76]. Most studies were conducted in Europe (*n* = 16, 50%) [48, 50, 53–56, 59, 61, 63, 64, 66, 70, 73, 77–79], followed by Asia (*n* = 6, 18.8%) [57, 60, 62, 67, 71, 77], North America (*n* = 4, 12.5%) [57, 58, 69, 72], South America (*n* = 4, 12.5%) [51, 52, 65, 68], and Australia (*n* = 1, 3.1%) [49]. One study included data from both Asia and North America (*n* = 1, 3.1%) [58].

Twenty-eight (87.5%) studies had a cross-sectional design [48–52, 54–59, 61–65, 67–75, 77–79], while four (12.5%) had a longitudinal design [53, 60, 66, 76]. For two of the longitudinal studies, however, only the cross-sectional baseline analyses were considered, as one did not provide analytic prospective associations [53] and the other reported only cross-sectional associations [60].

### Participants

Sample sizes ranged from 10 [59] to 1369 [57] participants, with a mean of 244 (standard deviation, SD 371) participants. On average, 67.5% of participants were women; ten studies (31.3%) included only women [48, 52, 55, 56, 61, 62, 67, 68, 70, 77], and two studies (6.3%) only men [59, 74]. Mean ages across study samples ranged from 65 (SD 17) [69] to 84 (SD 4) [74] years, with a pooled mean of 73.2 (SD 5.2) years.

Most studies included healthy, community-dwelling older adults with relatively high physical functioning, typically excluding individuals with neurological, musculoskeletal, cardiovascular, or metabolic disorders [49, 50, 52, 53, 57, 58, 61, 63, 64, 67, 69, 72, 74, 78, 79], limitations in functional mobility [50, 52–54, 56, 62, 66, 69, 70, 72, 74, 75, 77, 79], recent fractures or surgery [49, 58, 61, 65, 69, 70, 72], or conditions that might interfere with safe participation in mobility testing [49, 51, 64, 68, 73, 77]. Five studies (15.6%) excluded participants engaged in regular structured exercise [55, 64, 70, 72, 78]. Only two studies (6.3%) focused on specific populations: postmenopausal women with osteopenia or osteoporosis [70] and adults with mild disability or borderline sarcopenia [71].

Twenty-two of the 23 studies reporting status on locomotor capacity indicated that participants were highly functioning, reflected by mean SPPB scores of 10.5–11.6 pts [53, 56, 68, 69, 73], usual gait speeds of 1.0–1.2 m/s [51, 52, 74, 75, 78], TUG times of 5.8–10.3 s [62, 64, 76, 79], 6-Minute Walk Test (6MWT) distances of 556–567 m [50, 63, 67], a 5CST time of 7.2 (SD 1.8) s [71], a fast-pace TUG of 6.3 (SD 1.9) s [48], an 8-Foot Up-and-Go of 5.9 (SD 1.1) s [77], and by samples in which 82.5% of participants had usual-pace TUG of < 12 s [57] or 65% had usual gait speed of > 1.0 m/s [58]. Only one study included a sample with low locomotor capacity, indicated by a mean usual gait speed of < 0.8 m/s [66].

### Jump assessment

Jump performance was assessed using a countermovement jump (CMJ) in thirty-one studies (96.9%) [48–70, 72–79]. A small number of studies (also) employed alternative jump tests (3.1%), including squat jumps (SJs) [59, 68], single-legged hops [56], and a vertical jump without recoil [71].

#### Countermovement jumps

Testing procedures of CMJs varied considerably across the 31 studies conducting CMJs, particularly with regard to arm use, foot position, countermovement depth, number and selection of trials, rest intervals, and preparation procedures.

Most of these studies used either the hands-on-hips technique (*n* = 12; 38.7%) [48, 49, 51, 52, 55, 61, 65, 67, 68, 72, 73, 78] or did not specify arm use (*n* = 10; 32.3%) [50, 54, 57–60, 63, 64, 77, 79], while others (*n* = 8; 25.8%) allowed free arm use [53, 56, 62, 66, 69, 74–76], and one study (3.2%) required arms resting at the sides [70].

Only six studies (19.4%) specified initial foot position (shoulder-width [51, 52, 73, 78], hip-width [53], pelvis-width [48]), and just five (16.1%) reported details on the extent of countermovement (90° knee flexion [53, 59, 61], max. 70° controlled by apparatus [65], no restrictions [69]).

Nine-teen studies (61.3%) performed three CMJ trials [48–50, 53–55, 57–59, 61–64, 66, 67, 70, 72, 77, 78], while the twelve used two trials (*n* = 4; 12.9%) [71, 73, 76, 79], four to five trials (*n* = 2; 6.5%) [74, 75], three to five trials (n = 1; 3.2%) [65], three to four trials (*n* = 1; 3.2%) [56], at least 3 trials (*n* = 1; 3.2%) [69], or five trials (*n* = 1; 3.2%) [68], or did not report the number of CMJ trials (*n* = 2; 12.9%) [51, 52].

Rest intervals between CMJ trials ranged from 15 s [68] to 180 s [55] in the 15 studies (48.4%) that reported them [48, 50, 53–55, 61, 63–65, 68, 70, 72, 73, 78, 79], with a 60-s rest period being most common (*n* = 11; 35.5%) [50, 53, 54, 61, 63–65, 70, 72, 78, 79]. Two studies (6.5%) allowed self-selected rest intervals [56, 69], while 14 studies (45.2%) did not specify the rest period [49, 51, 57–60, 62, 66, 67, 71, 74–77].

For data analysis, nineteen studies (61.3%) used the trial with the maximum jump height [48, 50, 52, 57–59, 61, 63, 64, 66–70, 73–77]. Others used the trial with the maximum jump power (*n* = 6; 19.4%) [53–56, 78], the average of three trials (*n* = 2; 6.5%) [49, 65], or the last of three trials (*n* = 1; 3.2%) [62]. Three studies (9.7%) did not specify which trial was used for analysis [51, 60, 72].

Three studies (9.7%) reported additional preparation procedures, including a familiarization session a few days before testing [59], three calf raises as a warm-up [74], or a 5-min walk and jump-specific training one week prior to testing [68].

These variations in CMJ testing procedures may contribute to between-study heterogeneity and limit comparability across studies.

#### Alternative jumps

The two studies using the SJ did not specify arm use or foot position [59, 68]. One study predefined a 90° knee angle for the squat position [59], whereas the other did not report this information [68]. The number of trials also differed, with one study performing three trials with an unspecified rest interval [59] and the other conducting five trials with 15-s rest periods [68]. Both studies used the trial with the maximum jump height for data analysis and included a familiarization phase, either a few days before testing [59] or one week before testing combined with a 5-min warm-up walk before the SJs [68].

The vertical jump without recoil was performed from a standing position without specifications on arm use or foot placement. Two trials were completed, and the trial with the maximum jump height was used for analysis [71].

The single-legged hops were performed on the dominant or most comfortable leg with free arm use. Participants completed six to eight consecutive hops across three to four trials with self-selected rest intervals, and the hop with the maximum power was used for analysis; instructions emphasized forefoot hopping with a stiff knee and without heel contact [56].

#### Technical devices

Jump parameters were captured using a variety of technical devices. Twenty-six studies (81.3%) used force platforms [48–50, 53–59, 61–67, 69, 70, 72–75, 77–79].The most frequently used device was the Leonardo Mechanograph® force platform (Novotec Medical GmbH, Pforzheim, Germany), employed in 12 studies (37.5%) [48, 50, 54, 56–58, 62, 63, 66, 69, 70, 77]. Other force platforms included systems from Advanced Mechanical Technology Inc. (AMTI; Watertown, MA, USA) [49, 53, 72, 74, 75, 78, 79], Kistler Instrument Corp. (Amherst, NY, USA) [55, 61, 64, 65], MuscleLab (Stathelle, Norway)[73], Dinascan IBV [59], VISTI (St. Petersburg, Russia) [67], or a custom-built system [79].

Non-mechanography devices were used in six studies (18.7%) [51, 52, 60, 68, 71, 76]. Three studies used contact mats, which determine jump height from time-of-flight, including the Jump System Pro (CEFISE, São Paulo, Brazil) [51, 52] and the Elite Jump® (S2 Sports, São Paulo, Brazil) [68]. The other three studies used displacement-based digital vertical jump meters(Takei Scientific Instruments Co. Ltd., Niigata, Japan), which calculate jump height from the vertical extension of a tether connected to a waist belt [60, 71, 76].

#### Jump parameters

About half of the studies assessed a single jump parameter (*n* = 17; 53.1%) [49, 51–53, 55, 57–60, 67, 68, 71, 75–79], while the remaining studies reported multiple ones (*n* = 15; 46.9%) [48, 50, 54, 56, 61–66, 69, 70, 72–74].

Jump performance was most frequently quantified by jump power (*n* = 24, 75%), which was expressed as body-mass-normalized jump power (JP_norm_) in 18 studies [50, 53, 54, 56–58, 61–64, 66, 67, 69, 73–75, 77, 78], absolute jump power (JP_abs_) in four studies [55, 70, 72, 79], JP_norm_ and JP_abs_ in one study [48], or body-fat-normalized jump power and JP_abs_ in one study [65]. Jump power was calculated as peak power in 22 studies [48, 50, 53–58, 62–64, 66, 67, 69, 70, 73–75, 77–79], mean power in two studies [65, 72], and both in one study [61].

Jump height was reported in 17 studies (53.1%) [49, 51, 52, 54, 59–62, 64, 65, 68–73, 76].

Eight studies (25%) also assessed jump velocity, reported either as peak velocity [50, 54, 62, 63, 66, 69] or velocity at peak power [61, 74].

Jump force was measured in seven studies (21.9%), expressed as body-mass-normalized force [48, 50, 56, 61, 66, 74] and/or absolute force [48, 70]. Jump force was derived as peak force [48, 50, 56, 61, 66, 70]), mean force [61], or force at peak power [74].

Less commonly reported jump parameters included the Esslinger Fitness Index (defined as JP_norm_, adjusted for age and sex, and expressed as % of the sex-matched age-group mean) [54, 62], jump work [61], and jump impulse [65].

### Functional outcomes

Locomotor capacity was the most frequently assessed functional outcome, which was examined in 26 studies (81.3%) [48, 50–53, 55–57, 59–70, 72–75, 78, 79]. Other functional outcomes were assessed considerably less often: PA in four studies (12.5%) [48, 49, 71, 77], ADL functioning in three studies (9.4%) [48, 49, 54], and other function-related health outcomes in six studies (18.8%), including fall history [54, 57, 66], sarcopenia [54, 58], dysmobility syndrome [57, 58], and frailty [76].

#### Locomotor capacity

Of the 26 studies assessing locomotor capacity, 21 (80.8%) used multiple measures [48, 50–53, 56, 57, 60–66, 68, 69, 72–75, 79], whereas five (19.2%) relied on a single measure [55, 59, 67, 70, 78]. Locomotor capacity measures most frequently included the TUG (*n* = 15, 57.7%), performed with usual pace [53, 57, 62, 64, 66, 68, 73, 79] or fast pace [48, 50–52, 63, 65, 67], gait speed (*n* = 13, 50%) with usual pace [51, 53, 56, 60, 64, 66, 68, 69, 73–75, 78], fast pace [51, 72, 78], and/or under different conditions (e.g., usual, fast, dual-task, with obstacles) [78], the 5CST (*n* = 11, 42.3%) [51, 53, 56, 62, 66, 68, 69, 72–75].

Less commonly used locomotor capacity measures included the 6MWT (*n* = 5, 19.2%) [50, 51, 63, 67, 79], SPPB (*n* = 5, 19.2%) [53, 56, 68, 69, 73], One-Legged Stance (*n* = 3, 11.5%) [51, 60, 70], Functional Reach Test (*n* = 2, 7.7%) [60, 64], 400-m Walk Test (*n* = 2, 7.7%) [74, 75], posturography-based static and/or dynamic balance tests (*n* = 2, 7.7%) [59, 64], stair ascending and/or descending tests (*n* = 2, 7.7%) [61, 65], 30-Second Chair Stand Test (*n* = 1, 3.8%) [68], and a box-stepping test (*n* = 1, 3.8%) [55].

#### Physical activity

Objective monitoring was the primary approach in three of the four studies (75%) assessing PA, using either tri-axial accelerometers (ActiGraph GT3X) to quantify daily moderate-to-vigorous PA (MVPA) time or daily step counts [48, 77], or the doubly labeled water (DLW) method combined with a tri-axial accelerometer (Actimarker EW4800) to determine PA level (PAL_DLW_), total energy expenditure, daily MVPA and sedentary time, and daily step counts [71]. One study (25%) relied on self-reported activity using the International Physical Activity Questionnaire, expressed in metabolic equivalent (MET)-minutes per week [49].

#### Activities of daily living

ADL functioning was assessed in the three studies using different self-report questionnaires, including the ADL subscale of the Vertebral Fracture Study questionnaire [54], the independent living dimension of the Assessment of Quality of Life 8-Dimension instrument [49], and the Composite Physical Function scale [48].

#### Other function-related health outcomes

Fall history was assessed retrospectively over the previous 12 months in three studies [54, 57, 66]. Sarcopenia was evaluated in two studies, using either dual-energy X-ray absorptiometry to estimate appendicular lean mass [54] or the European Working Group on Sarcopenia in Older People 2 diagnostic criteria [58]. Dysmobility syndrome was examined in two studies, defined as the presence of at least three of six criteria (fall history, low handgrip strength, osteoporosis, low lean mass, high fat mass, and low locomotor capacity) [57, 58]. Frailty was assessed in one study using the Japanese adaptation of the Cardiovascular Health Study criteria [76].

### Risk of bias

The overall quality scores of the 31 cross-sectional studies ranged from 37.5% to 100%, with a mean score of 66.5% (SD 16.3). Most studies (*n* = 17, 54.8%) were rated as moderate quality (50–69%) [48–50, 52, 53, 55, 56, 60, 63–65, 67, 69, 70, 72, 78, 79], followed by 11 studies (35.5%) rated as high quality (≥ 70%) [51, 54, 57, 58, 66, 68, 71, 73–75, 77], and three studies (9.7%) rated as low quality (< 50%) [59, 61, 62]. The main sources of bias were insufficient reporting of sample and setting characteristics, unclear sample condition criteria, and the lack of identification or management of confounding factors. Detailed item-level ratings are presented in Supplementary Table S2.

The two longitudinal studies were both rated as moderate quality (50–69%), with overall scores of 63.6% [66] and 72.7% [76]. The main sources of bias were inadequate strategies to address potential confounding and incomplete reporting on dropout and follow-up. Detailed item-level ratings are presented in Supplementary Table S3.

### Meta-analysis of associations between jump performance and locomotor capacity

Meta-analyses were feasible only for locomotor capacity, as no other functional outcome category included at least three studies reporting statistically comparable associations for the same jump parameter and functional outcome measure. Only JP_norm_, jump height, and jump velocity, together with usual-pace TUG, fast-pace TUG, usual gait speed, 5CST, and SPPB, provided sufficient data to allow pooling. In total, 16 unique studies (50%) contributed data to at least one meta-analytic model [48, 50–52, 56, 60, 62–64, 66–69, 72, 73, 78].

#### Jump power

**Fig. 2:**
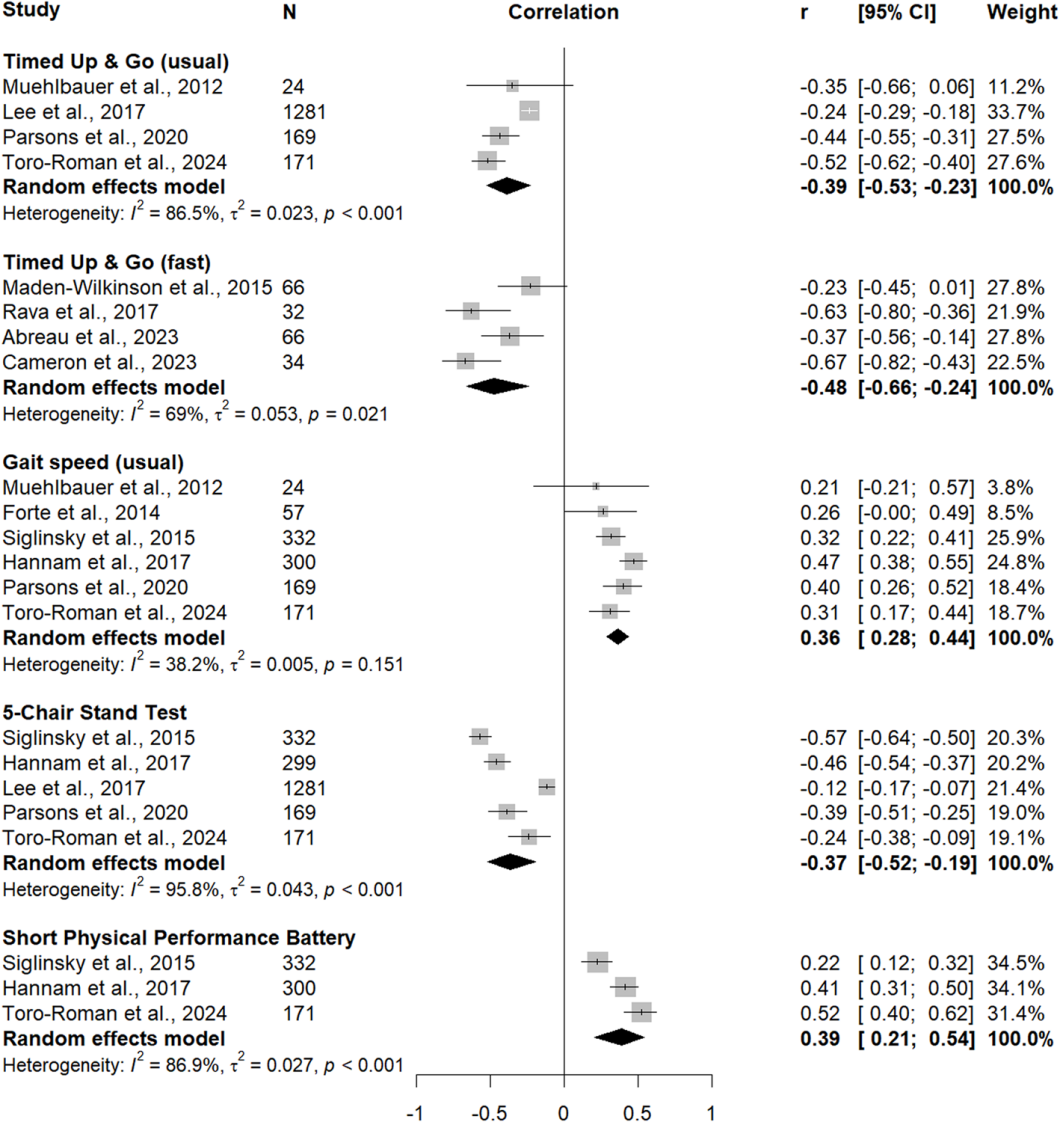
Random-effects meta-analysis of cross-sectional associations between jump power (normalized to body mass) and locomotor capacity outcomes

Associations between JP_norm_ and TUG were reported in eight studies, including 1645 participants for usual-pace TUG [62, 64, 66, 73] and 196 participants for fast-pace TUG [48, 50, 63, 67]. Pooling these studies resulted in moderate inverse correlations for both usual-pace TUG (*r*_*pooled*_ = –0.39, 95% confidence interval, CI –0.53, –0.23; I^2^ = 86.5%, Q-test *p* < 0.001) and fast-pace TUG (*r*_*pooled*_ = –0.48, 95% CI –0.66, –0.24; I^2^ = 69.0%, Q-test *p* = 0.021), each accompanied by high heterogeneity (Fig. 2).

Six studies (*n* = 1053) assessed associations between JP_norm_ and usual gait speed [56, 64, 66, 69, 73, 78]. The pooled analysis indicated a moderate positive correlation (*r*_*pooled*_ = 0.36, 95% CI 0.28, 0.44), with low-to-moderate heterogeneity (I^2^ = 38.2%, Q-test *p* = 0.151).

Pooling five studies (*n* = 2252) revealed a moderate inverse correlation for the association between JP_norm_ and 5CST (*r*_*pooled*_ = –0.37, 95% CI –0.52, –0.19), alongside high heterogeneity (I^2^ = 95.8%, *p* < 0.001) [56, 62, 66, 69, 73].

Three studies (*n* = 803) examined the association between JP_norm_ and SPPB, yielding a moderate positive correlation (*r*_*pooled*_ = 0.39, 95% CI 0.21, 0.54) with high heterogeneity (I^2^ = 86.9%, Q-test *p* < 0.001) [56, 69, 73].

#### Jump height

**Fig. 3:**
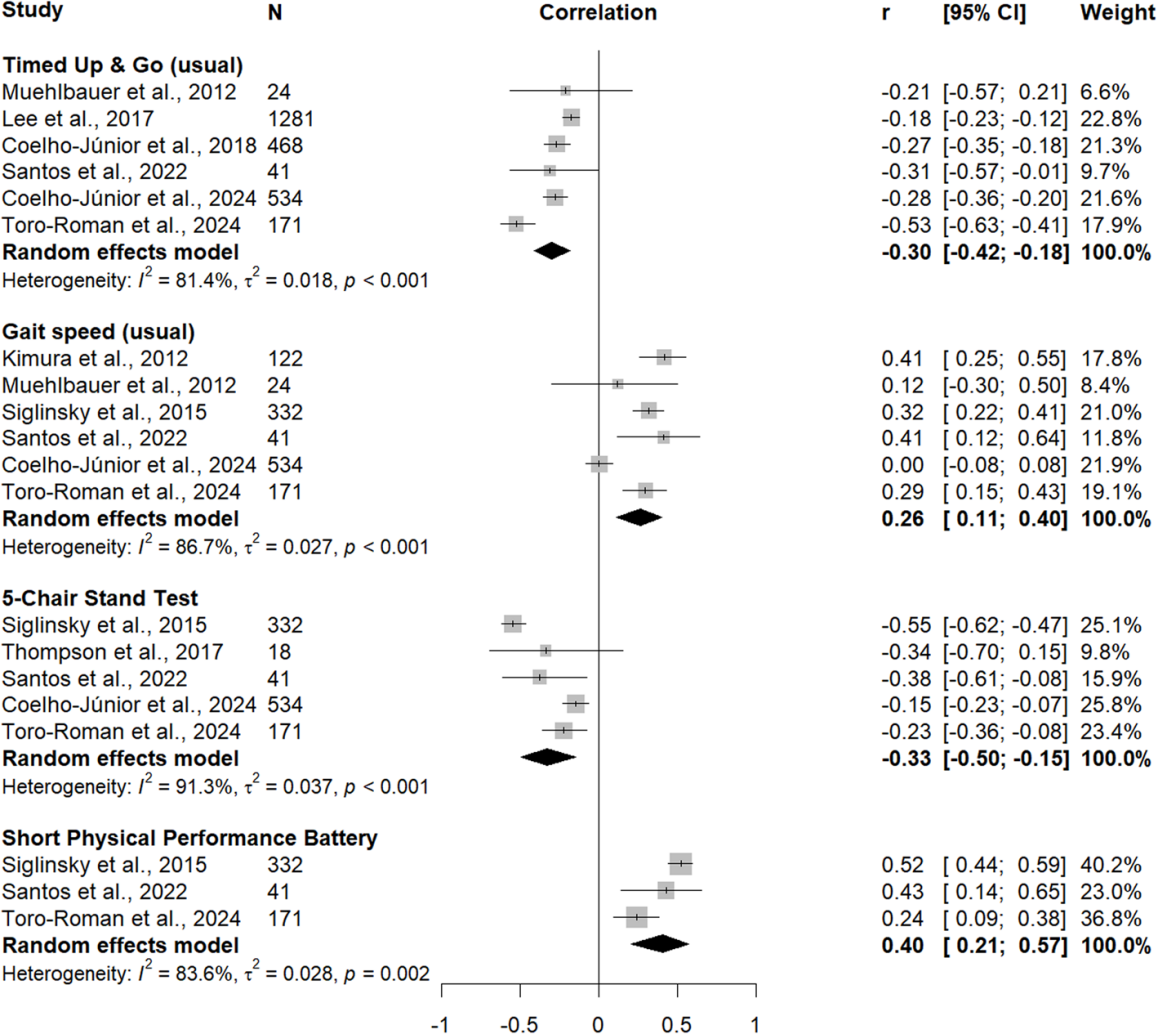
Random-effects meta-analysis of cross-sectional associations between jump height and locomotor capacity outcomes

Six studies (*n* = 2519) investigated the association between jump height and usual-pace TUG [51, 52, 62, 64, 68, 73], and the pooled findings showed a moderate inverse correlation (*r*_*pooled*_ = –0.30, 95% CI –0.42, –0.18), with high heterogeneity (I^2^ = 81.4%, Q-test *p* < 0.001) (Fig. 3).

The association between jump height and usual gait speed was examined in six studies (*n* = 1224) [51, 60, 64, 68, 69, 73]. The pooled analysis revealed a small positive correlation (*r*_*pooled*_ = 0.26, 95% CI 0.11, 0.40), with high heterogeneity (I^2^ = 86.7%, Q-test *p* < 0.001).

Pooling five studies (*n* = 1069) on associations between jump height and 5CST indicated a moderate inverse correlation (*r*_*pooled*_ = –0.33, 95% CI –0.50, –0.15), with high heterogeneity (I^2^ = 91.3%, Q-test *p* < 0.001) [51, 68, 69, 72, 73].

Three studies (*n* = 544) provided data on associations between jump height and SPPB, and the pooled analysis resulted in a moderate positive correlation (*r*_*pooled*_ = 0.40, 95% CI 0.21, 0.57), with high heterogeneity (I^2^ = 83.6%, Q-test *p* = 0.002) [68, 69, 73].

#### Jump velocity

**Fig. 4:**
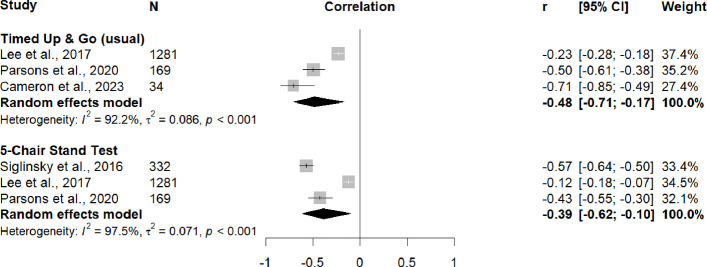
Random-effects meta-analysis of cross-sectional associations between jump velocity and locomotor capacity outcomes

Association between jump velocity and usual-pace TUG were reported in three studies (*n* = 1484) [50, 62, 66], and the pooled analysis showed a moderate inverse correlation (*r*_*pooled*_ = –0.48, 95% CI –0.71, –0.17) with high heterogeneity (I^2^ = 92.2%, Q-test *p* < 0.001). A moderate inverse correlation (*r*_*pooled*_ = –0.39, 95% CI –0.62, –0.10) with high heterogeneity (I^2^ = 97.5%, Q-test *p* < 0.001) was also identified across three studies (*n* = 1782) assessing the association between jump velocity and 5CST [62, 66, 69] (Fig. 4).

#### Subgroup analysis according to technological devices

Subgroup analyses were feasible only for associations between jump height and usual gait speed [51, 60, 64, 68, 69, 73] or usual-pace TUG [51, 52, 62, 64, 68, 73]. No significant differences were found between pooled correlations for jump height assessed using non-mechanography devices versus force platforms for usual gait speed (*r*_*pooled*_ = 0.27, 95% CI –0.03, 0.52 vs. 0.30, 95% CI 0.22, 0.38; Q-test *p* = 0.808) (Supplementary Figure S1) or usual-pace TUG (*r*_*pooled*_ = –0.33, 95% CI –0.55, –0.05 vs. –0.28, 95% CI –0.33, –0.22; Q-test *p* = 0. 710) (Supplementary Figure S2).

### Narrative synthesis of cross-sectional associations

#### Jump performance and physical activity

Of the four studies examining associations between jump performance and PA, two used adjusted models (age, sex, and/or body composition) [71, 77], while the other two reported unadjusted associations [48, 49].

Adjusted models consistently indicated that higher JP_norm_ or jump height was significantly associated with greater objectively measured PA (daily MVPA time, PAL_DLW_, total energy expenditure; (β_std_ = 0.34–0.37, *p* < 0.001–0.013) [71, 77].

Unadjusted analyses showed also significant moderate positive significant associations between jump power and daily step count (JP_norm_: *r* = 0.46; JP_abs_: *r* = 0.38) [48] or self-reported MET-minutes per week (*r* = 0.30) [49], whereas no significant associations were observed for jump force [48].

#### Jump performance and activities of daily living

One of the three studies on jump performance and ADL functioning used age-adjusted models [54], while two reported unadjusted associations [48, 49]. Age-adjusted models indicated that higher jump power, height or velocity, and a higher Esslinger Fitness Index were each significantly associated with fewer ADL impairments (*r*_adj_ = −0.28–0.38) [54]. Unadjusted analyses revealed significant moderate to large positive associations of jump power (JP_norm_: *r* = 0.58; JP_abs_: *r* = 0.49) [48] or jump height (*r* = 0.55) [49] with ADL functioning, whereas no significant associations were observed for jump force [48].

#### Jump performance and other function-related health outcomes

No significant associations were found between jump performance and previous 12-month fall history. One study reported no significant association between low JP_norm_ and prior falls in a model adjusted for demographic and anthropometric factors and multiple comorbidities (OR_adj_ = 1.43, 95% CI 0.98, 2.09) [57]. Similarly, another study observed no significant age-adjusted associations for JP_norm_ (OR_adj_ = 0.94, 95% CI 0.89, 1.00), jump height (OR_adj_ = 0.81, 95% CI 0.42,1.59), jump velocity (OR_adj_ = 0.93, 95% CI 0.79, 1.08), or the Esslinger Fitness Index (OR_adj_ = 0.98, 95% CI 0.96, 1.00) [54].

In contrast, low jump performance was significantly associated with sarcopenia. One study reported that low JP_norm_ was significantly associated with higher odds of having sarcopenia (OR_adj_ 4.07, 95% CI 1.45,11.41) in a model adjusted for demographic and anthropometric characteristics [58]. Another study found that lower jump power (OR_adj_ = 0.88, 95% CI 0.80, 0.96), jump velocity (OR_adj_ = 0.81, 95% CI 0.69, 0.97), jump height (OR_adj_ = 0.45, 95% CI 0.21, 0.96), and a lower Esslinger Fitness Index (OR_adj_ = 0.97, 95% CI 0.95, 0.99) were each significantly associated with higher odds of sarcopenia in age-adjusted models [54].

Significant associations were also reported between low jump performance and dysmobility syndrome. Low JP_norm_ was significantly associated with higher odds of dysmobility syndrome in models adjusted for demographic and anthropometric factors, with (OR_adj_ = 4.35, 95% CI 2.68, 7.09) [57] or without additional adjustment for multiple comorbidities (OR_adj_ = 4.32, 95% CI 2.40, 7.80) [58].

### Narrative synthesis of prospective associations

Prospective associations between jump performance and functional outcomes were analyzed in only two studies, both examining function-related health outcomes, including fall status [66] or frailty status [76]. In the study on fall status [66], lower JP_norm_ and lower jump velocity but not jump force were significantly associated with a higher likelihood of having experienced a fall in the previous 12 months at the 2–3-year follow-up in a model adjusted demographic and anthropometric factors. In contrast, in the study on frailty [76], no significant prospective association was observed between jump height and incident (pre-)frailty at the 5-year follow-up (OR_adj_ = 0.97, 95% CI 0.79, 1.19) in a model adjusted for sex and multiple physical and functional performance measures.

## Discussion

This systematic review and meta-analysis examined cross-sectional and prospective associations between jump performance and functional outcomes in older adults. Meta-analytic findings indicated consistent, moderate cross-sectional associations between higher jump power, height, or velocity and better locomotor capacity. These associations were predominantly observed in healthy, high-functioning community-dwelling older adults. Narrative synthesis further indicated that higher jump performance was cross-sectionally associated with greater PA, better ADL functioning, and lower odds of sarcopenia or dysmobility syndrome. In contrast, prospective associations were sparsely investigated and inconsistent; only two longitudinal studies were identified, suggesting that lower jump performance may be associated with future falls but not incident (pre-)frailty.

The observed moderate associations with locomotor capacity support the notion that jump performance captures an aspect of lower-limb neuromuscular capacity relevant for mobility-related function. The moderate magnitude of these associations suggests partial overlap with, but not redundancy to, commonly used locomotor capacity measures, consistent with jump performance reflecting a related yet distinct component of mobility-related function. Jump tests require rapid force generation and coordination in a demanding multi-joint movement, thereby challenging multiple mobility-relevant systems simultaneously. This may be particularly relevant in high-functioning older adults, in whom commonly used locomotor capacity measures, often involving less complex motor tasks, may be insufficiently challenging to detect subtle interindividual differences. Consistent with this interpretation, a single longitudinal study reported that jump parameters, but not the TUG, gait speed, or the 5CST, were independently associated with future falls in high-functioning older adults [66].

Beyond locomotor capacity, associations with PA, ADL functioning, or other function-related health outcomes were reported in only a small number of studies and varied in outcome definition and covariate adjustments. Findings for sarcopenia and dysmobility syndrome were promising but based on few studies, while no consistent cross-sectional associations were observed with fall history. Importantly, longitudinal evidence across all functional outcomes was scarce and heterogeneous, limiting conclusions about prospective associations between jump performance and functional decline over time. Given the predominance of cross-sectional designs, the available evidence should therefore be interpreted primarily as supporting convergent rather than predictive validity. Although one study suggested a prospective association with future falls [66], the small number of prospective analyses precludes generalization. Future research should prioritize prospective study designs to clarify whether jump performance is associated with incident mobility disability, decline in ADLs, falls, frailty progression, or other clinically relevant outcomes.

Between-study heterogeneity was generally high despite restricting meta-analyses to identical jump parameters and studies used largely comparable jump tests, with most of them assessing CMJs (96.9%) and using force platforms (87.5%). Residual heterogeneity likely reflects remaining variability in CMJ protocols, including differences in arm use, foot position, countermovement depth, number and selection of trial, rest intervals, and preparation procedures, many of which were inconsistently applied or insufficiently reported. These findings highlight the lack of standardized CMJ testing and reporting procedures, which has also been noted in younger populations [80], and underscore the need for greater methodological consistency to improve comparability and reduce heterogeneity in future studies. Accordingly, adoption of a standardized and well-documented CMJ protocol, such as a defined warm-up, hands-on-hips execution, three trials with 60-s rest intervals, and analysis of the trial with the maximum jump height, may improve comparability and facilitate evidence synthesis in future research. In addition, despite the predominance of healthy, high-functioning samples, differences in sex distribution, age range, and baseline physical functioning or activity levels may have further contributed to heterogeneity.

Jump power, height, and velocity showed similar moderate associations with locomotor capacity in the meta-analyses, with largely overlapping correlations and no parameter demonstrating stronger associations than another. This suggests that different jump parameters capture related aspects of jump performance associated with locomotor capacity rather than indicating the superiority of a single parameter. In contrast, jump force could not be meta-analyzed due to insufficient homogeneous data and, in studies examining jump force alongside jump height and/or jump power, generally showed weaker associations with locomotor capacity [50, 56, 61, 66, 70, 74], ADL functioning, and PA [48]. Similarly, in the limited longitudinal analyses available, jump power and velocity, but not jump force, showed prospective associations with future falls [66]. Although based on limited evidence, this pattern suggests that velocity-related aspects of jump performance are more closely associated with functional outcomes than force alone.

Jump performance was predominantly assessed using force platforms (87.5%), which allow detailed quantification of multiple jump parameters (e.g., power, height, velocity) but may be limited in routine or large-scale application due to cost and laboratory requirements. More accessible devices used in a minority of studies (12.5%), such as contact mats and displacement-based jump meters, captured jump height only. Importantly, associations with locomotor capacity were similar across jump parameters, and sensitivity analyses showed comparable associations between jump height measured with force platforms and non-mechanography devices. Together, these findings suggest that no measurement system is clearly superior and that also simpler approaches to assessing jump performance may capture functionally relevant aspects of mobility in older adults. Recent technological advances enable valid and reliable measurement of jump height using smartphone applications based on video-based motion analysis and machine learning [81, 82], as well as wrist-worn sport watches with an integrated inertial measurement [83]. These approaches may provide additional accessible and low-cost options for extending jump performance–based assessments beyond laboratory settings.

Several limitations should be considered when interpreting these findings. First, included studies were not specifically designed as measurement or validation studies of jump performance, but rather examined jump performance as part of broader observational or exploratory analyses. Consequently, this review synthesizes cross-sectional and prospective evidence derived from observational designs rather than formal psychometric evaluations, which would require different methodological frameworks and quality assessment tools. Second, the evidence base consisted primarily of cross-sectional studies, with only two longitudinal studies available, limiting conclusions regarding prospective associations between jump performance and functional outcomes. Third, substantial methodological and statistical heterogeneity was present across jump test protocols, functional outcome measures, and analytical approaches. Fourth, pooled estimates were based on unadjusted associations only and therefore do not account for potential confounding. Fifth, most studies included healthy, high-functioning older adults, limiting generalizability to more impaired or clinical populations. Sixth, study authors were not contacted to obtain additional information or clarify missing data, which may have introduced reporting bias and limited the completeness of the extracted data. Finally, inclusion was restricted to English and German publications, which may have introduced language bias.

## Conclusion

The findings of this review indicate that jump performance is consistently and moderately positively associated with locomotor capacity in high-functioning older adults, suggesting that it may have potential as a sensitive marker of early mobility-related functional decline. Evidence for cross-sectional associations with broader functional outcomes, such as PA, ADL, and other function-related health outcomes, as well as for prospective associations with functional decline, remains limited. Further research is needed to clarify associations between jump performance and non–locomotor capacity outcomes and, in particular, to examine prospective associations between jump performance and future functional outcomes in older adults.

## Supplementary Material

Supplementary Material 1

## Data Availability

All data used for the meta-analyses are provided in Supplementary Table S1.

## Abbreviations

5CST: 5-Chair Stand Test
ADL: Activities of daily living
CI: Confidence interval
CMJ: Countermovement jump
DLW: Doubly labeled water
JP_abs_: Absolute jump power
JP_norm_: Body-mass-normalized jump power
JBI: Joanna Briggs Institute
MET: Metabolic equivalent
MVPA: Moderate-to-vigorous physical activity
OR: Odds ratio
PA: Physical activity
PAL: Physical activity level
PRISMA: Preferred Reporting Items for Systematic Reviews and Meta-Analyses
RoB: Risk of bias
SD: Standard deviation
SJ: Squat jump
SPPB: Short Physical Performance Battery
TUG: Timed Up and Go
