# Supplementary material for: Cross-sectional and prospective associations between jump performance and functional outcomes in older adults: a systematic review and meta-analysis
Source: BMC Geriatr. 2026 Apr 11;26:533. doi: 10.1186/s12877-026-07450-6 (PMC13085499; doi:10.1186/s12877-026-07450-6)
Supplement: Supplementary file 1 — Supplementary Material 1 [file 12877_2026_7450_MOESM1_ESM.zip › Supplementary_table_S3_RoB_assessment_longitudinal.docx]

**Supplementarty Table S3.** Risk of bias assessment of the included longitudinal studies according to the Joanna Briggs Institute Checklist for Cohort Studies

| **Study** | **Item 1**  **Group similarity & recruitment** | **Item 2**  **Sample & setting** | **Item 3**  **Jump assessment** | **Item 4**  **Confounder identification** | **Item 5**  **Confounder management** | **Item 6**  **Baseline outcome- free** | **Item 7**  **Functional outcome measurement** | **Item 8**  **Adequate follow-up** | **Item 9**  **Follow-up completeness** | **Item 10**  **Dropout management** | **Item 11**  **Statistical analysis** | **Overall quality (%)** |
| --- | --- | --- | --- | --- | --- | --- | --- | --- | --- | --- | --- | --- |
| Parsons et al., 2020 | Yes ⚫ | Yes ⚫ | Yes ⚫ | Yes ⚫ | Yes ⚫ | Unclear ⚫ | Unclear ⚫ | Yes ⚫ | No ⚫ | No ⚫ | Yes ⚫ | Moderate (63.6%) |
| Yamagata et al., 2025 | Yes ⚫ | Yes ⚫ | Yes ⚫ | Yes ⚫ | Yes ⚫ | No ⚫ | Yes ⚫ | Yes ⚫ | No ⚫ | No ⚫ | Yes ⚫ | Moderate (72.7%) |
