# Supplementary material for: Cross-sectional and prospective associations between jump performance and functional outcomes in older adults: a systematic review and meta-analysis
Source: BMC Geriatr. 2026 Apr 11;26:533. doi: 10.1186/s12877-026-07450-6 (PMC13085499; doi:10.1186/s12877-026-07450-6)
Supplement: Supplementary file 1 — Supplementary Material 1 [file 12877_2026_7450_MOESM1_ESM.zip › Supplementary_table_S2_RoB_assessment_cross_sectional.docx]

**Supplementary Table S2.** Risk of bias Assessment of the included cross-sectional studies according to the Joanna Briggs Institute Checklist for Analytical Cross-Sectional Studies

| **Study** | **Item 1**  **Eligibility criteria** | **Item 2**  **Sample & setting** | **Item 3**  **Jump assessment** | **Item 4**  **Sample condition criteria** | **Item 5**  **Confounder identification** | **Item 6**  **Confounder management** | **Item 7**  **Functional outcome measurement** | **Item 8**  **Statistical analysis** | **Overall quality (%)** |
| --- | --- | --- | --- | --- | --- | --- | --- | --- | --- |
| Izquierdo et al., 1999 | No ⚫ | Unclear ⚫ | Yes ⚫ | No ⚫ | No ⚫ | No ⚫ | Yes ⚫ | Yes ⚫ | Low (37.5%) |
| Forte et al., 2008 | Yes ⚫ | Unclear ⚫ | Yes ⚫ | Yes ⚫ | No ⚫ | No ⚫ | Yes ⚫ | Yes ⚫ | Moderate (62.5%) |
| Larsen et al., 2009 | No ⚫ | Unclear ⚫ | Yes ⚫ | No ⚫ | No ⚫ | No ⚫ | Yes ⚫ | Yes ⚫ | Low (37.5%) |
| Kimura et al., 2012 | No ⚫ | Yes ⚫ | Yes ⚫ | No ⚫ | No ⚫ | No ⚫ | Yes ⚫ | Yes ⚫ | Moderate (50%) |
| Muehlbauer et al., 2012 | Yes ⚫ | Unclear ⚫ | Yes ⚫ | Yes ⚫ | No ⚫ | No ⚫ | Yes ⚫ | Yes ⚫ | Moderate (62.5%) |
| Forte et al., 2014 | Yes ⚫ | Unclear ⚫ | Yes ⚫ | Yes ⚫ | Unclear ⚫ | Unclear ⚫ | Yes ⚫ | Yes ⚫ | Moderate (62.5%) |
| Dietzel et al., 2015 | Yes ⚫ | Unclear ⚫ | Yes ⚫ | Unclear ⚫ | Yes ⚫ | Yes ⚫ | Yes ⚫ | Yes ⚫ | High (75%) |
| Maden-Wilkinson et al., 2015 | Yes ⚫ | Unclear ⚫ | Yes ⚫ | Unclear ⚫ | Unclear ⚫ | Unclear ⚫ | Yes ⚫ | Yes ⚫ | Moderate (50%) |
| Siglinsky et al., 2015 | Yes ⚫ | Unclear ⚫ | Yes ⚫ | Yes ⚫ | Unclear ⚫ | Unclear ⚫ | Yes ⚫ | Yes ⚫ | Moderate (62.5%) |
| Stenroth et al., 2015 | Unclear ⚫ | Unclear ⚫ | Yes ⚫ | Unclear ⚫ | Yes ⚫ | Yes ⚫ | Yes ⚫ | Yes ⚫ | Moderate (62.5%) |
| Rava et al., 2017 | Yes ⚫ | Unclear ⚫ | Yes ⚫ | Yes ⚫ | No ⚫ | No ⚫ | Yes ⚫ | Yes ⚫ | Moderate (62.5%) |
| Baldwin et al., 2017 | Yes ⚫ | Unclear ⚫ | Yes ⚫ | Yes ⚫ | No ⚫ | No ⚫ | Yes ⚫ | Yes ⚫ | Moderate (62.5%) |
| Hannam et al., 2017 | Unclear ⚫ | Yes ⚫ | Yes ⚫ | Yes ⚫ | Yes ⚫ | Yes ⚫ | Yes ⚫ | Yes ⚫ | Moderate (62.5%) |
| Lee et al., 2017 | Unclear ⚫ | Unclear ⚫ | Yes ⚫ | Unclear ⚫ | Unclear ⚫ | No ⚫ | Yes ⚫ | Yes ⚫ | Low (37.5%) |
| Thompson et al., 2017 | Yes ⚫ | Unclear ⚫ | Yes ⚫ | Yes ⚫ | No ⚫ | No ⚫ | Yes ⚫ | Yes ⚫ | Moderate (62.5%) |
| Coelho-Júnior et al., 2018 | Yes ⚫ | Unclear ⚫ | Yes ⚫ | Yes ⚫ | No ⚫ | No ⚫ | Yes ⚫ | Yes ⚫ | Moderate (62.5%) |
| Hong et al., 2018 | Yes ⚫ | Yes ⚫ | Yes ⚫ | Yes ⚫ | Yes ⚫ | Yes ⚫ | Yes ⚫ | Yes ⚫ | (100%) |
| Stolzenberg et al., 2018 | Yes ⚫ | Unclear ⚫ | Yes ⚫ | Yes ⚫ | Unclear ⚫ | No ⚫ | Yes ⚫ | Yes ⚫ | Moderate (62.5%) |
| Orssatto et al., 2020 | Unclear ⚫ | Unclear ⚫ | Yes ⚫ | Unclear ⚫ | Yes ⚫ | Yes ⚫ | Yes ⚫ | Yes ⚫ | Moderate (62.5%) |
| Takea et al., 2020 | Yes ⚫ | Unclear ⚫ | Yes ⚫ | Unclear ⚫ | Yes ⚫ | Yes ⚫ | Yes ⚫ | Yes ⚫ | High (75%) |
| Winger et al., 2020 | Yes ⚫ | Yes ⚫ | Yes ⚫ | Yes ⚫ | Yes ⚫ | Yes ⚫ | Yes ⚫ | Yes ⚫ | High (100%) |
| Hong et al., 2021 | Unclear ⚫ | Yes ⚫ | Yes ⚫ | Yes ⚫ | Yes ⚫ | Yes ⚫ | Yes ⚫ | Yes ⚫ | High (87.5%) |
| Winger et al., 2021 | Yes ⚫ | Unclear ⚫ | Yes ⚫ | Yes ⚫ | Yes ⚫ | Yes ⚫ | Yes ⚫ | Yes ⚫ | High (87.5%) |
| Santos et al., 2022 | Yes ⚫ | Yes ⚫ | Yes ⚫ | Yes ⚫ | No ⚫ | No ⚫ | Yes ⚫ | Yes ⚫ | High (75%) |
| Zymbal et al., 2022 | Yes ⚫ | Unclear ⚫ | Yes ⚫ | Yes ⚫ | Yes ⚫ | Yes ⚫ | Yes ⚫ | Yes ⚫ | High (87.5%) |
| Abreu et al., 2023 | Yes ⚫ | Unclear ⚫ | Yes ⚫ | Unclear ⚫ | No ⚫ | No ⚫ | Yes ⚫ | Yes ⚫ | Moderate (50%) |
| Cameron et al., 2023 | Yes ⚫ | Yes ⚫ | Yes ⚫ | Unclear ⚫ | No ⚫ | No ⚫ | Yes ⚫ | Yes ⚫ | Moderate (62.5%) |
| Coelho-Júnior et al., 2024 | Yes ⚫ | Yes ⚫ | Yes ⚫ | Unclear ⚫ | Yes ⚫ | Yes ⚫ | Yes ⚫ | Yes ⚫ | High (87.5%) |
| Toro-Román et al., 2024 | Yes ⚫ | Unclear ⚫ | Yes ⚫ | Unclear ⚫ | Yes ⚫ | Yes ⚫ | Yes ⚫ | Yes ⚫ | High (75%) |
| Parsons et al., 2020 | Unclear ⚫ | Yes ⚫ | Yes ⚫ | Unclear ⚫ | Yes ⚫ | Yes ⚫ | Yes ⚫ | Yes ⚫ | High (75%) |
| Diekmann et al., 2022 | Yes ⚫ | Unclear ⚫ | Yes ⚫ | Yes ⚫ | No ⚫ | No ⚫ | Yes ⚫ | Yes ⚫ | Moderate (62.5%) |
