# Supplementary material for: Cross-sectional and prospective associations between jump performance and functional outcomes in older adults: a systematic review and meta-analysis
Source: BMC Geriatr. 2026 Apr 11;26:533. doi: 10.1186/s12877-026-07450-6 (PMC13085499; doi:10.1186/s12877-026-07450-6)
Supplement: Supplementary file 1 — Supplementary Material 1 [file 12877_2026_7450_MOESM1_ESM.zip › Supplementary_table_S1_study_characteristics.docx]

**Supplementary Table S1.** Study characteristics

| **Study (country)** | **Study design** | **Participants** | | | **Jump Assessment** | | | **Functional outcome** | **Cross-sectional & prospective associations** |
| --- | --- | --- | --- | --- | --- | --- | --- | --- | --- |
|  |  | **Sample size**  **(% women)** | **Mean (SD) age, years^a^** | **Key characteristics** | **Jump test** | **Technical device** | **Jump parameter** |  |  |
| Izquierdo et al., 1999 (Spain) | Cross-sectional | 10 (0) | 71 (5) | Inclusion: n/a  Exclusion: n/a  Healthy, habitually physically active; no prior experience in regular strength training or competitive sports; no use of medications expected to affect physical performance | CMJ: countermovement to 90° knee flexion; 3 trials; jump with maximum height used for analysis  SJ: 90° knee angle squat; 3 trials; jump with maximum height used for analysis  (familiarization session a few days before actual jump tests) | Dinascan IBV 600M force platform (Instituto de Biomecánica de Valencia, Valencia, Spain) | Jump height [cm] | Locomotor capacity (dynamic leg-stance balance test [posturography]) | **Jump height** [cm] (CMJ)  CoP sway area: *r* = -0.46  CoP sway path: *r* = 0.78**  **Jump height** [cm] (SJ)  CoP sway area: *r* = -0.05  CoP sway path: *r* = 0.53* |
| Forte et al., 2008 (Italy) | Cross-sectional | 32 (100) | 68.8 (2.8) | Inclusion: medically stable  Exclusion: practiced no more than one hour twice a week  Community-dwelling | CMJ: hands on hips; 3 trials with 180-s rest; jump with maximum power used for analysis | Kistler Type 9261 force platform (Kistler Instruments AG, Winterthur, Switzerland) | Jump power [W] (peak) | Locomotor capacity (box-stepping test) | **Jump power** [W]  Box-stepping test: *r* = 0.75** |
| Larsen et al., 2009 (Denmark) | Cross-sectional | 17 (100) | 72.4 (6.4) | Inclusion: n/a  Exclusion: n/a  Community-dwelling; moderately trained (~ 60 min exercise once a week); no orthopedic or neurological disorders; no lower-limb fracture within past 5 years | CMJ: hands on hips; countermovement to ~90° knee flexion; 3 trials with 60-s rest; jump with maximum height used for analysis | Kistler Type 9281B force platform (Kistler Instrumente AG, Winterthur, Switzerland) | Jump power [W/kg] (peak, mean)  Jump velocity [m/s] (at peak power)  Jump force [N/kg] (peak, mean)  Jump work [J/kg]  Jump height [cm] | Locomotor capacity (stair ascending) | **Jump power** [W/kg] (peak / mean)  Stair ascending: *r* = 0.745***^b^ / 0.690**^b^  **Jump velocity** [m/s] (at peak power)  Stair ascending: *r* = 0.881***^b^  **Jump force** [N/kg] (peak / mean / at peak power)  Stair ascending: *r* = 0.161^b^ / 0.330^b^ / 0.100^b^  **Jump work** [J/kg]  Stair ascending: *r* = 0.837***^b^  **Jump height** [cm]  Stair ascending: *r* = 0.765***^b^ |
| Kimura et al., 2012 (Japan) | Longitudinal (cross-sectional analyses only) | 122 (57.4) | 73.1 (5.8)^c^ | Inclusion: ≥60 years, healthy  Exclusion: n/a  Community-dwelling | CMJ: 2 trials (several familiarization trials) | JUMP-MD TKK5106 Digital Vertical Jump Meter (Takei Scientific Instruments Co. Ltd., Niigata, Japan) | Jump height [cm] | Locomotor capacity (GS, FRT, OLS) | **Jump height** [cm]  GS (usual-pace): *r* = -0.414^d^  FRT: *r* = 0.327^c^  OLS (eyes open): *r* = 0.422^d^ |
| Muehlbauer et al., 2012 (Germany) | Cross-sectional | 24 (54.2) | 70.3 (4.6) | Inclusion: ≥65 years, independent community-dwelling, naïve to strength, power, and balance training  Exclusion: musculoskeletal, neurological, or orthopedic disorders that affected ability to perform strength, power, or balance tests; any medical conditions limiting participation (e.g. terminal illness, cognitive dysfunction)  Mean TUG = 8.2 (SD 1.3) s  Mean GS (usual-pace) = 1.3 (SD 0.2) m/s | CMJ: 3 trials with 60-s rest; jump with maximum height used for analysis | Kistler Type 9281A force platform (Kistler Instrumente AG, Winterthur, Switzerland) | Jump power [W/kg] (peak)  Jump height [cm] | Locomotor capacity (TUG, GS, FRT, static/proactive balance test [posturography]) | **Jump power** [W/kg]  TUG (usual-pace): *r* = -0.353  GS (usual-pace): *r* = 0.214 (sign-corrected)  FRT: *r* = 0.137  Static balance: CoP_AP_ sway path: *r* = 0.199; CoP_ML_ sway path: *r* = 0.214  Proactive balance: SO_AP_: *r* = 0.026; SO_ML_: *r* = 0.013  **Jump height** [cm]  TUG (usual-pace): *r* = -0.330  GS (usual-pace): *r* = 0.117 (sign-corrected)  FRT: *r* = 0.268  Static balance: CoP_AP_ sway path: *r* = 0.111; CoP_ML_ sway path: *r* = 0.375  Proactive balance: SO_AP_: *r* = -0.173; SO_ML_: *r* = 0.123 |
| Forte et al., 2014 (Ireland) | Cross-sectional | 57 (57.9) | 69.4 (3.3)^c^ | Inclusion: 65-75 years  Exclusion: >1 exercise session/week; fall in past 2 years, musculoskeletal or neurological diseases; severe arthritis; cardiac, cerebrovascular, or uncontrolled metabolic disease  Community-dwelling  Mean GS (usual-pace) = 1.2 (SD 0.3) m/s | CMJ: hands on hips; feet shoulder-width apart; 3 trials with 60-s rest; jump with maximum power used for analysis | AMTI Model BP400600-2000 force platform (Advanced Mechanical Technology Inc., Watertown, MA, USA) | Jump power [W/kg] (peak) | Locomotor capacity (GS) | **Jump power** [W/kg]  GS (usual-pace): *r* = 0.260*  GS (fast-pace): *r* = 0.275*  GS (dual-task): *r* = 0.100  GS (picking-up): *r* = 0.418***  GS (package carrying): *r* = 0.444***  GS (obstacle): *r* = 0.508***  GS (15-cm corridor): *r* = 0.371**  **Jump power** [W/kg] independently associated with 15-cm corridor GS (*β_std_* = 0.336 *p* = 0.016), obstacle GS (*β_std_* = 0.379, *p* = 0.005), and package-carrying GS (*β_std_* = 0.396, *p* = 0.006), after adjustment for muscle strength, postural control, and gender. |
| Dietzel et al., 2015 (Germany) | Cross-sectional | 293 (49.8) | 71.9 (7.4)^c^ | Inclusion: 60-85 years  Exclusion: inability to walk unaided; invalid body composition estimates due to metal implants, prostheses, edema, or medications affecting water-mineral homeostasis; refusal of X-ray exposure; inability to understand study procedures and follow instructions  Community-dwelling | CMJ: 3 trials with 60-s rest; jump with maximum power used for analysis | Leonardo Mechanograph Ground Reaction Force Plate (Novotec Medical GmbH, Pforzheim, Germany) | Jump power [W/kg] (peak)  Jump velocity [m/s] (peak)  Jump height [cm]  Esslinger Fitness Index [%]  (jump power [W/kg], adjusted for age and sex, expressed as % of the sex-matched age-group mean) | ADL (subscale of the Vertebral Fracture Study questionnaire)  Sarcopenia (DXA)  Fall history (self-report; last 12 months) | **Jump power** [W/kg] (crude / age-adjusted)  ADL: *r* = -0.375^d^ / *r_adj_* = -0.278^d^  Sarcopenia: OR_crude_ = 0.89 (95% CI 0.83, 0.95)^e^ / OR_adj_ = 0.88 (95% CI 0.80, 0.96)^e^  Fall history: OR_crude_ = 0.94 (95% CI 0.89, 1.00)^e^ / OR_adj_ = 0.98 (95% CI 0.91, 1.05)^e^  **Jump velocity** [m/s] (crude / age-adjusted)  ADL: *r* = -0.361^d^ / *r_adj_* = -0.260^d^  Sarcopenia: OR_crude_ = 0.81 (95% CI 0.71, 0.93)^e^ / OR_adj_ = 0.81 (95% CI 0.68, 0.97)^e^  Fall history: OR_crude_ = 0.79 (95% CI 0.77, 0.98)^e^ / OR_adj_ = 0.94 (95% CI 0.81, 1.10)^e^  **Jump height** [cm] (crude / age-adjusted)  ADL: *r* = -0.347^d^ / *r_adj_* = -0.244^d^  Sarcopenia: OR_crude_ = 0.41 (95% CI 0.23, 0.76)^e^ / OR_adj_ = 0.41 (95% CI 0.18, 0.98)^e^  Fall history: OR_crude_ = 0.58 (95% CI 0.34, 0.98)^e^ / OR_adj_ = 0.82 (95% CI 0.48, 1.41)^e^  **Esslinger Fitness Index** [%]  ADL: *r* = -0.284^d^  Sarcopenia: OR_adj_ = 0.97 (95% CI 0.95, 0.99)^e^  Fall history: OR_adj_ = 0.98 (95% CI 0.96, 1.00)^e^ |
| Maden-Wilkinson et al., 2015 (United Kingdom) | Cross-sectional | 66 (53.0) | 72 (5) | Inclusion: n/a  Exclusion: pregnant or breastfeeding women; musculoskeletal and metabolic diseases  Community-dwelling; healthy; socially active; no medical contraindications  Mean 6MWT = 546 (SD 75) m^b^ | CMJ: 3 trials with 60-s rest; jump with maximum height used for analysis | Leonardo Mechanograph Ground Reaction Force Plate (Novotec Medical GmbH, Pforzheim, Germany) | Jump power [W/kg] (peak)  Jump velocity [m/s] (peak) | Locomotor capacity (TUG, 6MWT) | **Jump power** [W/kg]  TUG (fast-pace): *r* = -0.23^d^  6MWT: *r* = 0.35^d^  **Jump velocity** [m/s]  TUG (fast-pace): *r* = -0.27^d^  6MWT: *r* = 0.38^d^ |
| Siglinsky et al., 2015 (USA) | Cross-sectional | 332 (64.1) | 65.4 (17.4) | 4 cohorts: (1) persons aged 25-74 years: *n* = 122; (2) postmenopausal women aged 47-83 years: *n* = 52; (3) older persons aged ≥70 years: *n* = 62; (4) older persons aged ≥70 years *n* = 96  Inclusion: community-dwelling, >25 years (2 cohorts ≥70 years)  Exclusion (varied by cohort): generally excluded those with serious illness, recent surgery or cardiovascular event, cognitive impairment, inability to walk or stand unaided, very low bone mineral density (T-score < −3.5 with prior fracture), high vitamin D (>30 ng/mL), or recent cancer  Mean SPPB = 10.5 (SD 1.5) pt | CMJ: free arm use; no restrictions in degree of countermovement; ≥3 trials with self-chosen rest periods; jump with maximum height used for analysis | Leonardo Mechanograph Ground Reaction Force Plate (Novotec Medical GmbH, Pforzheim, Germany) | Jump power [W/kg] (peak)  Jump velocity [m/s] (peak)  Jump height [cm] | Locomotor capacity (GS, SPPB, 5CST) | **Jump power** [W/kg]  GS (usual-pace): *r* = 0.316^b^  SPPB: *r* = 0.520^b^  5CST: *r* = 0.574^b^  **Jump velocity** [m/s]  GS (usual-pace): *r* = 0.346^b^  SPPB: *r* = 0.529^b^  5CST: *r* = 0.600^b^  **Jump height** [cm]  GS (usual-pace): *r* = 0.316^b^  SPPB: *r* = 0.520^b^  5CST: *r* = 0.548^b^ |
| Stenroth et al., 2015 (Finland) | Cross-sectional | 52 (50.0) | 74.8 (3.3) | Inclusion: n/a  Exclusion: n/a  Community-dwelling; moderately socially active; free from major diseases; no mobility limitations (able to walk 250 m unassisted); medically stable  Mean TUG = 6.7 (SD 1.2) s | CMJ: 2 trials with 60-s rest; jump with maximum power used for analysis | Custom-built force platform | Jump power [W] (peak) | Locomotor capacity (TUG, 6MWT) | **Jump power** [W]  TUG (usual-pace): *r_adj_* = -0.419**  6MWT: *r_adj_* = 0.443**  (adjusted for sex, age, body mass, and height)  **Jump power** [W] not independently with 6MWT associated (*β_std_* = 0.321, *p* = 0.098), when adjusted for sex, age, body mass, height, plantarflexor muscle-tendon properties, and leg muscle strength. |
| Rava et al., 2017 (Korea) | Cross-sectional | 32 (100) | 71.9 (4.4) | Inclusion: ≥65 years; healthy, independent community-dwelling; weight stable over last 6 months  Exclusion: cardiac or neurological illness; joint replacement, or any other condition interfering with motor function  Mean 6MWT = 567 (SD 89) m | CMJ: 3 trials (rest not specified); jump with maximum height used for analysis | PD-3A force platform (VISTI, Russia) | Jump power [W/kg] (peak) | Locomotor capacity (TUG) | **Jump power** [W/kg]  TUG (fast-pace): *r* = -0.663***^b^ |
| Baldwin et al., 2017 (Australia) | Cross-sectional | 300 (50.0) | Range 60-101 (50 participants per 10-year interval) | Inclusion: healthy; able to perform ADLs  Exclusion: major physical disability; insufficient English, health conditions affecting physical performance (e.g., prior joint replacement/major surgery, diabetes, cancer, neurological disorders, severe cardiac, pulmonary, or musculoskeletal disease); mobility-aid dependence; BMI ≥ 40; pregnancy  Community-dwelling | CMJ: hands on hips; 3 trials; average of the 3 trials used for analysis | AMTI force platform (Advanced Mechanical Technology Inc., Watertown, MA, USA) | Jump height [cm] | Activity of daily living (AQoL-8D independent living)  Physical activity (IPAQ) | **Jump height** [cm]  IPAQ [MET-mins] (*n* = 297): *r* = 0.296**  AQoL-8D independent living): *r* = 0.546** |
| Hannam et al., 2017 (United Kingdom) | Cross-sectional | 300 (100) | 76.4 (2.6) | Inclusion: born 1927-1942; living in Bristol/Avon and contactable in 2015; community-dwelling; SPPB ≥6 pt.; able to produce valid jump/hop data  Exclusion: none  Community-dwelling  Mean SPPB = 10.6 (SD 1.4) pt. | CMJ: free arm use; 3-4 trials with self-chosen rest intervals; jump with maximum power used for analysis  Single-legged hops: dominant/most comfortable leg; free arm use; 6-8 hops; 3-4 trials with self-chosen rest; jump with maximum power used for analysis (“hop on your forefoot, without landing on your heels using a stiff knee”) | Leonardo Mechanograph Ground Reaction Force Plate (Novotec Medical GmbH, Pforzheim, Germany) | CMJ: Jump power [W/kg] (peak)  Hops: Jump force [N/kg] (peak) | Locomotor capacity (GS, 5CST, SPPB) | **Jump power** [W/kg] (crude / adjusted for age, height, comorbidities)  GS (usual-pace): *r* = 0.47** / *β_std_* = 0.44 (95% CI 0.18, 0.70)** (sign reversal)  5CST: *r* = -0.46** / *β_std_* = -0.42 (95% CI -0.70, -0.13)** (*n* = 299)  SPPB: *r* = 0.41* / *β_std_* = 0.39 (95% CI 0.08, 0.70)**  **Jump force** [N/kg] (crude / adjusted for age, height, comorbidities)  GS (usual-pace): *r* = 0.13 / *β_std_* = 0.13 (95% CI 0.05, 0.21)** (sign reversal)  5CST: *r* = -0.24** / *β_std_* = -0.23 (95% CI -0.34, -0.11) (*n* = 299)  SPPB: *r* = 0.22** / *β_std_* = 0.22 (95% CI 0.12, 0.32)** |
| Lee et al., 2017 (Korea) | Cross-sectional | 1281 (100) | 71.0 (4.4) | Inclusion: n/a  Exclusion: unable to perform jumping test  Community-dwelling  Mean TUG = 10.3 (SD 3.9) s | CMJ: free arm use; 3 trials; last jump that was technically adequate was recorded | Leonardo Mechanograph Ground Reaction Force Plate (Novotec Medical GmbH, Pforzheim, Germany) | Jump power [W/kg] (peak)  Jump velocity [m/s] (peak)  Jump height [cm]  Esslinger Fitness Index [%] | Locomotor capacity (TUG, 5CST) | **Jump power** [W/kg]  TUG (usual-pace): *r* = -0.237***  5CST: *r* = -0.120***  **Jump velocity** [m/s]  TUG (usual-pace): *r* = -0.233***  5CST: *r* = -0.124***  **Jump height** [cm]  TUG (usual-pace): *r* = -0.177***  5CST: r = -0.076**  **Esslinger Fitness Index** [%]  TUG (usual-pace): *r* = -0.207***  5CST: *r* = -0.144*** |
| Thompson et al., 2017 (USA) | Cross-sectional | 18 (55.6) | 71.1 (5.9) | Inclusion: 65-85 years; able to walk without assistive device; BMI 19-33 kg/m^2^  Exclusion: hip/knee joint surgery in past year; neuromuscular disease; regular engagement in structured exercise; uncontrolled hypertension; other medical reasons for not engaging in exercise  Community-dwelling | CMJ: hands on hips; 3 trials with 60-s rest | AMTI AccuPower force platform (Advanced Mechanical Technology Inc., Watertown, MA, USA) | Jump power [W] (mean)  Jump height [cm] | Locomotor capacity (GS, 5CST) | **Jump power** [W]  10-m GS (fast-pace): *r* = 0.69  400-m GS (fast-pace): *r* = 0.71  5CST: *r* = -0.22  **Jump height** [cm]  10-m GS (fast-pace): *r* = 0.71**  400-m GS (fast-pace): *r* = 0.81**  5CST: *r* = -0.34  **Jump height** [cm] independently associated with 400-m GS (*β_std_* = -0.807, p <0.001) but not with 10-m GS or 5CST, after adjustment for single- and multiple joint peak torque |
| Coelho-Júnior et al., 2018 (Brazil) | Cross-sectional | 468 (100) | 65.8 (6.0) | Inclusion: ≥60 years; community-dwelling; female; ADL independent; cognitively intact; ambulatory without assistive devices  Exclusion: institutionalized; major cardiorespiratory; neurological, psychiatric, musculoskeletal, or vestibular disorders; controlled comorbidities permitted  Mean GS (usual-pace) = 1.2 (SD 0.2) m/s | CMJ: hands on hips; feet shoulder-width apart; jump with maximum height used for analysis | Jump System Pro contact mat (CEFISE, São Paulo, Brazil) | Jump height [cm] | Locomotor capacity (TUG) | **Jump height** [cm]  TUG (fast-pace): *r* =-0.270* |
| Hong et al., 2018 (Korea) | Cross-sectional | 1369 (66.2) | 71.6 (4.4) | Inclusion: ≥65 years; community-dwelling  Exclusion: living in the current residence <8 months; planning to move within 2 years; active inflammatory, leukocytosis, current malignancy, or incomplete data  Low TUG (≥12 s): *n* = 239 (17.5%) | CMJ: 3 trials; jump with maximum height used for analysis | Leonardo Mechanograph^®^ Ground Reaction Force Plate (Novotec Medical GmbH, Pforzheim, Germany) | Jump power [W/kg] (peak) | Dysmobility syndrome (≥3 of 6 factors: fall history, low handgrip strength, osteoporosis, low lean mass, high fat mass, and low locomotor capacity)  Locomotor capacity (TUG)  Fall history (self-report; last 12 months) | **Jump power** [W/kg] (group into tertiles or failed-to-jump)  Dysmobility syndrome:  Mid vs. high: OR_crude_ = 2.13 (95% CI 1.34, 3.38)**  Low vs. high: OR_crude_ = 5.55 (95% CI 3.62, 8.50)***  Failed-to-jump vs. high: OR_crude_ = 11.30 (95% CI 6.78, 18.82)***  Mid vs. high: OR_adj_ = 1.90 (1.17, 3.11)*  Low vs. high: OR_adj_ = 4.35 (2.68, 7.09)***  Failed-to-jump vs. high: OR_adj_ = 7.60 (4.16, 13.89)***  Low TUG (≥12 s):  Mid vs. high: OR_adj_ = 1.05 (95% CI 0.64, 1.71)  Low vs. high: OR_adj_ = 2.64 (95% CI 1.67, 4.21)***  Failed-to-jump vs. high: OR_adj_ = 5.13 (95% CI 2.90, 9.08)***  Fall history:  Mid vs. high: OR_adj_ = 1.05 (95% CI 0.73, 1.51)  Low vs. high: OR_adj_ = 1.43 (95% CI 0.98, 2.09)  Failed-to-jump vs. high: OR_adj_ = 2.02 (95% CI 1.21, 3.35)**  (OR_adj_ = adjusted for age, sex, BMI, malnutrition, chronic pain, depressive symptoms, physical inactivity, serum albumin level, and presence of degenerative arthritis) |
| Stolzenberg et al., 2018 (Germany) | Cross-sectional | 63 (100) | 66.6 (4.1) | Inclusion: total hip or lumbar spine (L1-L4) T-score from -2.0 to -3.0 SD on dual-energy X-ray absorptiometry; able to walk without any aids  Exclusion: participation in strength/balance exercise in past 6 months; testing-apparatus experience; walking aid use; bone-active medication; venous thrombosis <2 years; joint inflammation <6 months; tendinopathy; recent fracture (<12 months); metal implant/pacemaker; neuromuscular or balance disorder; rheumatoid arthritis; smoking >20 cigarettes/day; uncontrolled hypertension  Post-menopausal women with osteopenia or osteoporosis | CMJ: arms resting at sides; 3 trials with 60-s rest; jump with maximum height used for analysis | Leonardo Mechanograph^®^ Ground Reaction Force Plate (Novotec Medical GmbH, Pforzheim, Germany) | Jump power [W] (peak)  Jump force [N] (peak)  Jump height [cm] | Locomotor capacity (OLS) | **Jump power** [W]  OLS time (unstable surface, eyes open): *r* = 0.29*  OLS CoP area (stable surface, eyes open): *r* = -0.27*  OLS CoP velocity or area (stable surface, eyes open or closed): range *r* = -0.18 to -0.06  **Jump force** [N]  OLS time (unstable surface, eyes open): *r* = 0.09  OLS CoP area or velocity (stable surface, eyes open or closed): range *r* = -0.19 to -0.08  **Jump height** [cm]  OLS time (unstable surface, eyes open): *r* = 0.56***  OLS CoP area or velocity (stable surface, eyes open or closed): range *r* = -0.14 to -0.02  **Jump power** [W] independently associated with OLS CoP area (stable surface, eyes open) (*p* = 0.014) and **jump height** [cm] independently associated with OLS time (unstable surface, eyes open) (*p* < 0.001), after adjustment for age; height; weight; jump height, force, and/or power; leg-press 10-repetition maximum, calf muscle area and calf muscle density as covariates |
| Orssatto et al., 2020 (Brazil) | Cross-sectional | 24 (33.3) | 66.4 (4.7) | Inclusion: n/a  Exclusion: n/a  No lower limb musculoskeletal disorders; no fractures or orthopedic surgery in the last 5 years  Mean TUG (fast-pace) = 5.6 (SD 0.7) s | CMJ: hands on hips; countermovement to max. 70° knee flexion controlled by apparatus; 3-5 trials with 60-s rest; average of 3 trials with lowest variation used for analysis | Kistler Quattro Jump force platform (Kistler Instrument Corp., Amherst, NY, USA) | Jump power [W/kg, W] (mean; normalized to body fat)  Jump impulse [N⋅s/kg, N⋅s] (normalized to body fat)  Jump height [cm] | Locomotor capacity (TUG, stair ascending & descending duration) | **Jump power** [W/kg]  TUG (fast-pace): *r* = n/a  Stair ascending / descending: *r* = -0.64** / -0.60**  **Jump power** [W]  TUG (fast-pace): *r* = -0.45*  Stair ascending / descending: *r* = n/a / -0.46*  **Jump impulse** [N⋅s/kg]  TUG (fast-pace): *r* = n/a  Stair ascending / descending: *r* = -0.65** / -0.61**  **Jump impulse** [N⋅s]  TUG (fast-pace): *r* = -0.47*  Stair ascending / descending: *r* = n/a / -0.47*  **Jump height** [cm]  TUG (fast-pace): *r* =-0.55**  Stair ascending / descending: *r* = -0.57** / -0.68*** |
| Takea et al., 2020 (Japan) | Cross-sectional | 54 (70.4) | 71.9 (6.9) | Inclusion: mild disability defined as borderline dementia (Hasegawa Dementia Scale<12 pt.), borderline sarcopenia (GS <1 m/s, handgrip strength <25 kg men / <20 kg women), underweight or overweight, type-2 diabetes, hyperlipemia, or hypertension  Exclusion: n/a  Community-dwelling  Mean 5CST = 7.2 (SD 1.8) s | Vertical jump: jump from standing position without recoil: 2 trials; jump with maximum height used for analysis | JUMP-MD TKK5406 Digital Vertical Jump Meter (Takei Scientific Instruments Co. Ltd., Niigata, Japan) | Jump height [cm] | Physical activity (DLW method, Actimarker EW4800) | **Jump height** [cm]  PAL_DLW_: *r* = 0.414^b^  Daily MVPA time: *r* = 0.485^b^  Daily sedentary time: *r* = -0.285 (only women)  Daily steps: *r* = 0.477^b^  **Jump height** [cm] independently associated with PAL_DLW_ (*β_std_ =* 0.367, *p* = 0.013) and total energy expenditure (*β_std_ =* 0.340, *p <* 0.001), after adjustment for body composition and sex. |
| Winger et al., 2020 (USA) | Cross-sectional | 1242 (0) | 84 (4) | Inclusion: ≥65 years; ability to walk unassisted; ability to provide self-reported data and informed consent; residence near a clinical site  Exclusion: bilateral hip replacement or any severe disease/condition that would result in imminent death  Community-dwelling  Mean GS (usual-pace) = 1.18 (SD 0.22 m/s) | CMJ: free arm use; 3 trials (max. 4-5 trials if ≥ 1/3 jumps had data quality or technical problems); 3 calf rises as warm-up; jump with highest jump height, or highest peak jump power if all trials were without flight, used for analysis | AMTI AccuPower force platform (Advanced Mechanical Technology Inc., Watertown, MA, USA) | Jump power [W/kg] (peak)  Jump velocity [m/s] (at peak power)  Jump force [N/kg] (at peak power) | Locomotor capacity (GS, 400-m walk time, 5CST) | **Jump power** [W/kg]  GS (usual-pace): *r_adj_* = 0.44***  400-m walk time: *r_adj_* = -0.51***  5CST: *r_adj_* = -0.47***  **Jump velocity** [m/s]  GS (usual-pace): *r_adj_* = 0.41***  400-m walk time: *r_adj_* = -0.45***  5CST: *r_adj_* = -0.43***  **Jump force** [N/kg]  GS (usual-pace): *r_adj_* = 0.20***  400-m walk time: *r_adj_* = -0.29***  5CST: *r_adj_* = -0.27***  (*r_adj_* = adjusted for age, race, site, and height; jump velocity additionally weight-adjusted)  **Jump power** [W/kg] (\|*β_std_*\| *=* 0.42-0.47, all *p* < 0.05), **jump velocity** [m/s] (\|*β_std_*\| *=* 0.37-0.42, all *p* < 0.05), and **jump force** [N] (\|*β_std_*\| *=* 0.18-0.24, all *p* < 0.05) independently associated with GS (usual-pace), 400-m walk time, and 5CST, after adjustment for age, race, site, height, falls history, blood pressure, hip/joint pain, bone mineral density, executive functioning, diabetes, hypertension, congestive heart failure, myocardial infarction, stroke, Parkinson’s Disease, and total number of medications. |
| Hong et al., 2021 (Korea, USA) | Cross-sectional | 285 (68.4) | 75.0 (3.9) | 2 cohorts:  Inclusion: n/a (cohort 1 & 2)  Exclusion: active inflammatory, leukocytosis; current malignancy; refused or failed to jump (cohort 1); recent cardiovascular events; major surgery; severe end-organ disease; history of malignancy with metastasis to the musculoskeletal system; unable to stand without assistance (cohort 2)  Community-dwelling  Low gait speed (<1.0 m/s): *n* = 101 (35%) | CMJ: arm use, foot position, and countermovement not specified; 3 trials; jump with maximum height used for analysis | Leonardo Mechanograph^®^ Ground Reaction Force Plate (Novotec Medical GmbH, Pforzheim, Germany) | Jump power [W/kg] (peak) | Sarcopenia (EWGSOP2)  Dysmobility syndrome (≥3 of 6 factors: fall history, low handgrip strength, osteoporosis, low lean mass, high fat mass, and low locomotor capacity) | **Jump power** [W/kg]  Sarcopenia:  Low vs. high: OR_crude_ = 4.30 (1.61, 11.45)**  Low vs. high: OR_adj_ = 4.07 (1.45, 11.41)**  Dysmobility syndrome:  Low vs. high: OR_crude_ = 5.41 (3.11, 9.40)***  Low vs. high: OR_adj_ = 4.32 (2.40, 7.80)***  (OR_adj_ = adjusted for age, sex, height, and ethnicity) |
| Winger et al., 2021 (USA) | Cross-sectional | 56 (55.4) | 79.3 (5.9) | Inclusion: ≥70 years  Exclusion: self-reported health contraindication to physical testing and the inability to perform basic mobility tasks (e.g. severe pain, aching, or stiffness while walking), cognitive impairment  Community-dwelling  Mean GS (usual-pace) = 1.2 (SD 0.2) m/s | CMJ: free arm use; 3 trials (max. 4-5 trials if ≥ 1/3 jumps had data quality or technical problems); 3 calf rises as warm-up; jump with highest jump height, or highest peak jump power if all trials were without flight, used for analysis | AMTI AccuPower force platform (Advanced Mechanical Technology Inc., Watertown, MA, USA) | Jump power [W/kg] (peak) | Locomotor capacity (GS, 400-m walk time, 5CST) | **Jump power** [W/kg]  GS (usual-pace): *r_adj_* = 0.31*  400-m walk time: *r_adj_* = -0.54*  5CST: *r_adj_* = 0.27  (*r_adj_* = adjusted for age, race, site, and height)  **Jump power** [W/kg] independently associated with GS (usual-pace), 400-m walk time, and 5CST (all *p* < 0.05), after adjustment for age, sex, race, height, self-reported diabetes, self-reported peripheral vascular disease and self-reported pain in legs/feet |
| Diekmann et al., 2022 (Germany) | Longitudinal (only cross-sectional analyses included) | 176 (60.2) | 75.3 (3.7) | Inclusion: ≥70 years; community-dwelling; ability to attend assessments independently; TUG < 20 s  Exclusion: severe acute diseases; difficulties in climbing a flight of 10 steps; pacemaker or other electronic implants  Mean SPPB = 11.0 (SD 1.0) pt. | CMJ: free arm use; feet hip-width apart; countermovement to ~90° knee flexion; 3 trials with 60-s rest; jump with maximum power used for analysis | AMTI AccuPower force platform (Advanced Mechanical Technology Inc., Watertown, MA, USA) | Jump power [W] (peak) | Locomotor capacity (TUG, GS, 5CST, SPPB, 6MWT, SCPT) | **Jump power** [W]  TUG (usual-pace): *r* = -0.10^f^  GS (usual-pace): *r* = 0.23**^f^  5CST: *r* = 0.14^f^  SPPB: *r* = -0.10^f^  6MWT: *r* = 0.29**^f^  SCPT: *r* = 0.67**^f^ |
| Santos et al., 2022 (Brazil) | Cross-sectional | 41 (100) | 71.0 (6.2) | Inclusion: ≥60 years  Exclusion: medical contraindication for moderate-intensity exercise; dementia; pain/discomfort during physical assessments  Community-dwelling  Mean SPPB = 11.6 (SD 0.6) pt. | CMJ: hands on hips; 5 trials with 15-s rest; jump with maximum height used for analysis  SJ: 5 trials with 15-s rest; jump with maximum height used for analysis  (5-min walk before jump tests to activate muscles, jumping training 1 week before for familiarization) | Elite Jump^®^ contact mat (S2 Sports, São Paulo, Brazil) | Jump height [cm] | Locomotor capacity (TUG, GS, SPPB, 5CST, 30s-CST) | **Jump height** [cm] (CMJ)  TUG (usual-pace): *r* = -0.314*  GS (usual-pace): *r* = 0.410**  5CST: *r* = -0.378*  SPPB: *r* = 0.426**  30s-CST: *r* = 0.412**  **Jump height** [cm] (SJ)  TUG (usual-pace): *r* = -0.314*  GS (usual-pace): n/a  5CST: *r* = -0.471**  30s CST: *r* = 0.498******* |
| Zymbal et al., 2022 (Portugal) | Cross-sectional | 54 (100) | 73.5 (6.8) | Inclusion: ≥65 years; community-dwelling  Exclusion: inability to perform the tests due to severe health or mobility problems (inability to stand or walk alone)  Mean 8-Foot Up and Go = 5.9 (SD 1.1) s | CMJ: 3 trials; jump with maximum height used for analysis | Leonardo Mechanograph Ground Reaction Force Plate (Novotec Medical GmbH, Pforzheim, Germany) | Jump power [W/kg] (peak) | Physical activity (Actigraph, GT3X) | **Jump power** [W/kg]  Daily MVPA time: *β_std_* = 0.36*  (adjusted for age) |
| Abreu et al., 2023 (Portugal) | Cross-sectional | 66 (100) | 73.6 (8.2) | Inclusion: ≥65 years, community-dwelling  Exclusion: inability to perform physical tests; health issues  Mean TUG (fast-pace) = 6.3 (SD 1.9) s | CMJ: hands on hips; feet pelvis-width apart; 3 trials with 30-s rest; jump with maximum height used for analysis | Leonardo Mechanograph Ground Reaction Force Plate (Novotec Medical GmbH, Pforzheim, Germany) | Jump power [W/kg, W]  (peak)  Jump force [N/kg, N] (peak) | Locomotor capacity (TUG)  ADL (CPF scale)  Physical activity (Actigraph GT3X) | **Jump power** [W/kg]  TUG (fast-pace): *r* = -0.37***  CPF scale: *r* = 0.58***  Daily steps: *r* =0.46***  **Jump power** [W]  TUG (fast-pace): *r* = -0.47***  CPF scale: *r* = 0.49***  Daily steps: *r* = 0.38***  **Jump force** [N/kg]  TUG (fast-pace): *r* = -0.01  CPF scale: *r* = 0.16  Daily steps: *r* = 0.14  **Jump force** [N]  TUG (fast-pace): *r* = -0.19  CPF scale: *r* = 0.06  Daily steps: *r* = 0.39 |
| Cameron et al., 2023 (United Kingdom) | Cross-sectional | 34 (50.0) | 71.2 (3.5)^c^ | Inclusion: community-dwelling; medically stable  Exclusion: institutionalization; unable to walk 250 m unassisted; major co-morbidities (cardiac, metabolic, pulmonary, neurological); recent hip/knee replacement or immobilization  Mean 6MWT = 551 (SD 72) m^b^ | CMJ: 3 trials with 60-s rest; jump with maximum height used for analysis | Leonardo Mechanograph Ground Reaction Force Plate (Novotec Medical GmbH, Pforzheim, Germany) | Jump power [W/kg] (peak)  Jump velocity [m/s] (peak)  Jump force [N/kg] (peak) | Locomotor capacity (TUG, 6MWT) | **Jump power** [W/kg]  TUG (fast-pace): *r* = -0.67***  6MWT: *r* = 0.56***  **Jump force** [N/kg]  TUG (fast-pace): *r* = 0.375*  6MWT: *r* = 0.151  **Jump velocity** [m/s]  TUG (fast-pace): *r* = -0.71***  6MWT: *r* = 0.57***  (values derived from figures) |
| Coelho-Júnior et al., 2024 (Brazil) | Cross-sectional | 534 (88.4) | 67.6 (6.3) | Inclusion: ≥60 years, self-sufficient; able to complete all study measurements as outlined in the protocol  Exclusion: n/a  Community-dwelling  Mean GS (usual-pace) = 1.0 (SD 2.7) m/s | CMJ: hands on hips; feet shoulder-width apart | Jump System Pro contact mat (CEFISE, São Paulo, Brazil) | Jump height [cm] | Locomotor capacity (TUG, GS, 5CST, 6MWT, OLS) | **Jump height** [cm] (crude / adjusted for age, BMI, sex, comorbidities, 5CST duration, and 5CST absolute, relative, and allometric muscle power)  TUG (fast-pace): *r* = -0.28*** / *β* = -0.04 (95% CI -0.06, -0.02)***  GS (usual-pace): *r* = 0.00 / *β* = 0.00 (95% CI -0.06, -0.06)  GS (fast-pace): *r* = 0.08 / *β* = 0.02 (95% CI -0.07, 0.01)  5CST: *r* = -0.15*** / n/a  6MWT: *r* = 0.13*** / *β* = 3.77 (95% CI 1.17, 6.37)**  OLS: *r* = 0.18*** / *β* = 0.07 (95% CI -0.13, 0.28) |
| Toro-Román et al., 2024 (Spain) | Cross-sectional | 171 (63.2) | 72.7 (6.1) | Inclusion: ≥60 years; independent in physical testing; living in the study region  Exclusion: medical contraindication for exercise  Community-dwelling  Mean SPPB = 11.2 (SD 1.2) pt.^b^ (sex distribution similar across age groups) | CMJ: hands on hips; feet shoulder-width apart; 2 trials with 30-s rest; jump with maximum height used for analysis | MuscleLab force platform (MuscleLab, Stathelle, Norway) | Jump power [W/kg] (peak)  Jump height [cm] | Locomotor capacity (TUG, GS, 5CST, SPPB) | **Jump power** [W/kg]  TUG (usual-pace): *r* = -0.521***  GS (usual-pace): *r* = 0.309***  5CST: *r* = -0.240**  SPPB: *r* = 0.222**  **Jump height** [cm]  TUG (usual-pace): *r* = -0.528***  GS (usual-pace): *r* = 0.295***  5CST: *r* = -0.226**  SPPB: *r* = 0.241** |
| Parsons et al., 2020 (United Kingdom) | Longitudinal  (follow-up = 2-3 years) | 169 (~50) | 75.1 (2.5) | Inclusion: born 1931-2004 in Hertfordshire; still living there in 1998-2004; able to jump  Exclusion: n/a  Community-dwelling  Mean GS (usual-pace) = 0.80 (SD 0.15) m/s | CMJ: free arm use; 3 trials; jump with maximum height used for analysis | Leonardo Mechanograph Ground Reaction Force Plate (Novotec Medical GmbH, Pforzheim, Germany) | Jump power [W/kg] (peak)  Jump velocity [m/s] (peak)  Jump force [N/kg] (peak) | Locomotor capacity (TUG, GS, 5CST)  Fall history (self-report; last 12 months) | Cross-sectional associations with locomotor capacity  **Jump power** [W/kg]  TUG (usual-pace): *r* = -0.44**  GS (usual-pace): *r* = 0.40**  5CST: *r* = -0.39**  **Jump velocity** [m/s]  TUG (usual-pace): *r* = -0.50**  GS (usual-pace): *r* = 0.46**  5CST: *r* = -0.43**  **Jump force** [N/kg]  TUG (usual-pace): *r* = -0.09  GS (usual-pace): *r* = 0.04  5CST: *r* = -0.13  Prospective associations with fall status at 2 to 3-year follow-up  **Jump power** [W/kg]  OR_crude_ = 0.91 (95% CI 0.85, 0.98)  **Jump velocity** [m/s]  OR_crude_ = 0.20 (95% CI 0.05, 0.72)  **Jump force** [N/kg]  OR_crude_ = n/a  (ORs were robust to adjustments for age, height, and sex; detailed information not provided) |
| Yamagata et al., 2025 (Japan) | Longitudinal (follow-up: 5 years) | 52 (61.5) | 72.5 (4.6) | Inclusion: ≥65 years  Exclusion: n/a  Community-dwelling  Mean TUG = 5.8 (SD 0.9) s^g^ | CMJ: free arm use; 2 trials (several familiarization trials); jump with maximum height used for analysis | JUMP-MD TKK5406 Digital Vertical Jump Meter (Takei Scientific Instruments Co. Ltd., Niigata, Japan) | Jump height [cm] | Frailty (Japanese version of the CHS criteria) | **Jump height** [cm]  (Pre-)Frailty: OR_adj_ = 0.97 (95% CI 0.79, 1.19)  (adjusted for sex, grip strength, 30s-CST, TUG, Fitness Age Score, Kihon Checklist oral function) |

Abbreviations: 30s-CST, 30-second Chair Stand Test; 5CST, Five-Chair Stand Test; 6MWT, 6-Minute Walk Test; ADL, activities of daily living; AQoL-8D, Assessment of Quality of Life (8 dimensions); CHS, Cardiovascular Health Study; CI, confidence interval; CMJ, countermovement jump; CoP, center of pressure; CoP_AP_, center of pressure in anterior-posterior direction; CoP_ML_, center of pressure in medio-lateral direction; CPF scale, Composite Physical Function scale; DLW, doubly labeled water; DXA, dual-energy X-ray absorptiometry; EWGSOP2, European Working Group on Sarcopenia in Older People 2; FRT, Functional Reach Test; GS, gait speed; IPAQ, International Physical Activity Questionnaire; MET-min, metabolic equivalent minutes; MVPA, moderate-to-vigorous physical activity; OLS, one-leg stance; OR, odds ratio; OR_adj_, adjusted odds ratio; OR_crude_, unadjusted odds ratio; PAL, physical activity level; PAL_DLW_, physical activity level assessed by doubly labeled water; *r*, Pearson correlation coefficient; *r*_adj_, adjusted Pearson correlation coefficient; SCPT, Stair Climb Power Test; SD, standard deviation; SJ, squat jump; SO_AP_, summed platform oscillations in anterior-posterior direction; SO_ML_, summed platform oscillations in medio-lateral direction; SPPB, Short Physical Performance Battery; TUG, Timed Up and Go; *β_std_*, standardized beta coefficient.

^a^ unless otherwise specified.

^b^ Correlation coefficient (r) derived from reported *R*^2^ using r = √R² (sign determined by direction of association in text or figure).

^c^ Pooled (sample-size-weighted) mean and standard deviation calculated from male and female subgroups.

^d^ Pooled (sample-size-weighted) correlation coefficient (*r*) calculated from male and female subgroups using Fisher’s z-transformation and back-transformed to *r*.

^e^ Pooled (sample-size-weighted) odds ratio (OR) calculated from male and female subgroups using natural-log transformation and back-transformed to OR.

^f^ Correlation coefficients (r) transformed from Spearman’s *ρ* using Fisher’s approximation (r = 2·sin(π·ρ / 6)).

^g^ Pooled (sample-size-weighted) mean and standard deviation calculated from robust and frailty-risk subgroups.

* *p* < 0.05, ** *p* < 0.01*, *** *p* < 0.001.
