# Supplementary material for: Cross-sectional and prospective associations between jump performance and functional outcomes in older adults: a systematic review and meta-analysis
Source: BMC Geriatr. 2026 Apr 11;26:533. doi: 10.1186/s12877-026-07450-6 (PMC13085499; doi:10.1186/s12877-026-07450-6)
Supplement: Supplementary file 1 — Supplementary Material 1 [file 12877_2026_7450_MOESM1_ESM.zip › Supplementarty_figures_S1_S2_meta_analysis_technical_device.docx]

**Supplementarty Figure S1.** Random-effects meta-analysis of cross-sectional associations between jump height and usual gait speed, stratified by technical device.


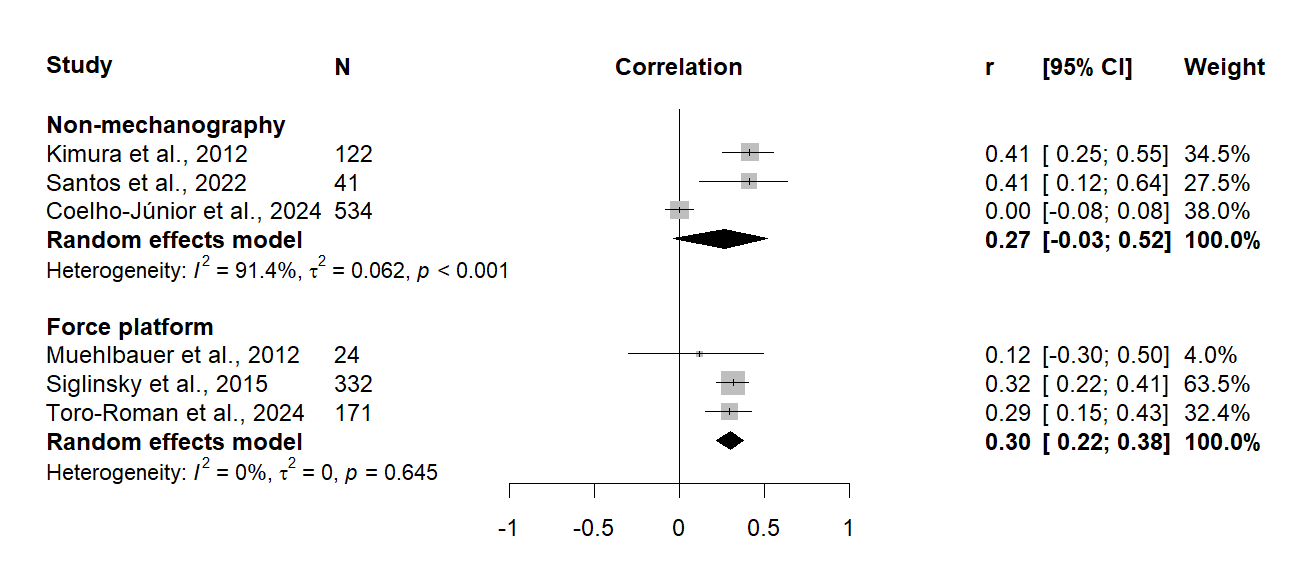


**Supplementarty Figure S2.** Random-effects meta-analysis of cross-sectional associations between jump height and Timed Up and Go, stratified by technical device.

**
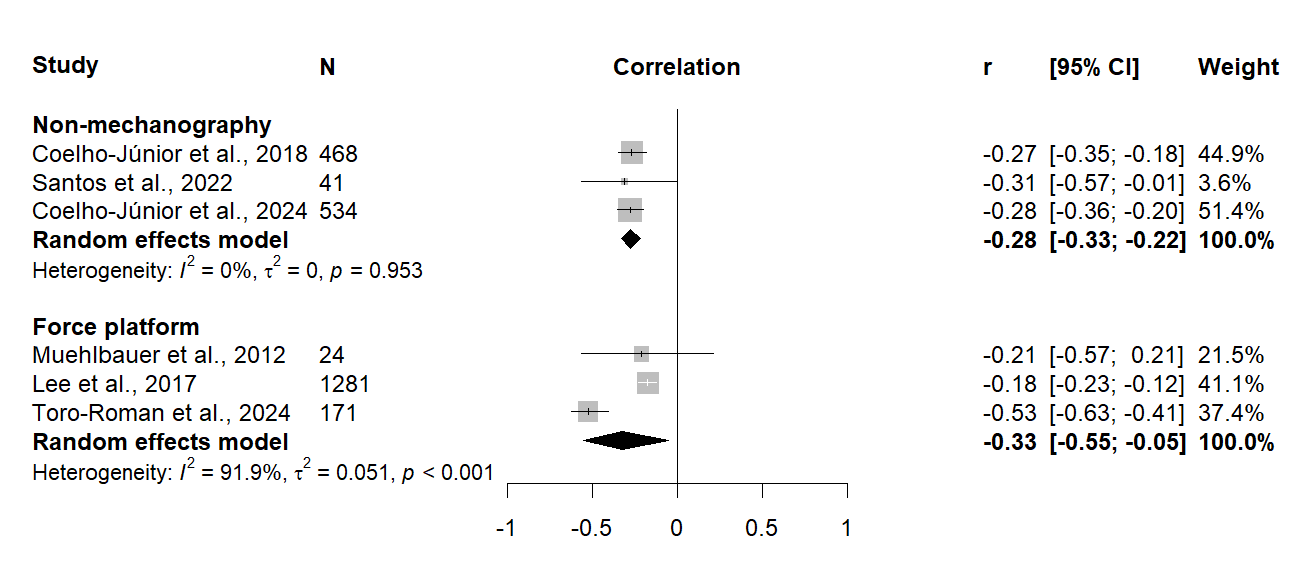
**
